# Cox10-mediated mitochondrial respiration in brown adipocytes regulates adaptive thermogenesis and systemic metabolism

**DOI:** 10.1016/j.isci.2026.116442

**Published:** 2026-06-26

**Authors:** Esther Paulo, Yuanyuan Wu, Biao Wang

**Affiliations:** 1 Cardiovascular Research Institute, Department of Physiology, University of California, San Francisco, San Francisco, CA 94158, USA

**Keywords:** human metabolism, molecular biology, metabolic flux analysis

## Abstract

Mitochondrial respiration is essential for Ucp1-mediated thermogenesis in brown adipocytes, where heat production depends on oxygen-driven mitochondrial activity. To define the role of complex IV, we generated brown-adipocyte-specific Cox10-knockout mice (Cox10^BKO^), as Cox10 is required for cytochrome *c* oxidase assembly. Cox10-deficient brown adipocytes exhibited markedly reduced complex IV activity and impaired Ucp1-dependent thermogenesis. Although ATF4 signaling was strongly induced, the alternative ATF4-dependent thermogenic pathway failed due to suppression of global protein synthesis, consistent with severe mitochondrial stress and reduced ribosomal gene expression. Unexpectedly, Cox10^BKO^ mice housed at room temperature or thermoneutrality were protected against high-fat-diet-induced obesity and insulin resistance. These findings demonstrate that brown adipocytes regulate systemic metabolic homeostasis independently of canonical thermogenic function and suggest that respiration-deficient brown fat may promote metabolic fitness through endocrine or metabolic signaling mechanisms.

## Highlights

• Cox10 enables complex IV respiration in brown adipocyte
• Cox10 loss impairs Ucp1-dependent adaptive thermogenesis
• ATF4 activation fails due to suppressed protein synthesis
• Cox10BKO mice resist obesity despite defective thermogenesis

## Graphical abstract

**Figure:**
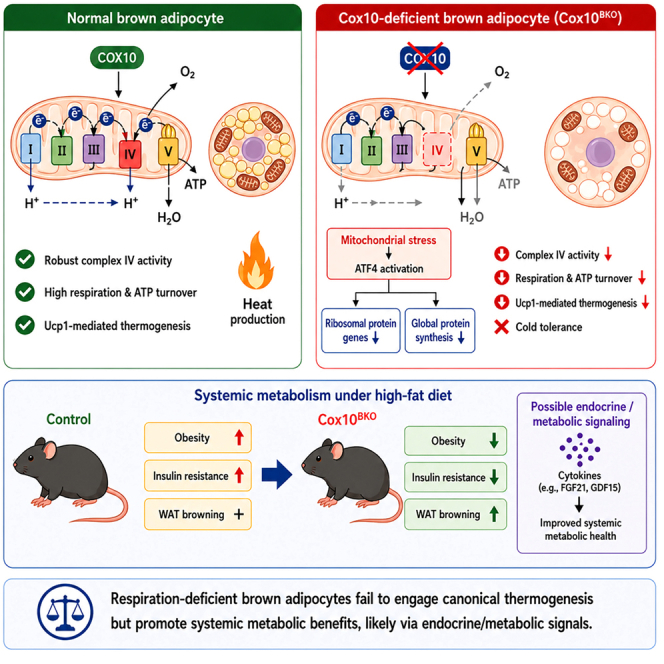

## Introduction

Thermogenesis, the heat-producing process through obligatory basal metabolism and facultative responses to environmental challenges such as shivering from muscle and brown adipose tissue (BAT)-mediated non-shivering thermogenesis, enables endotherms to maintain a stable core body temperature.^1^^,^^2^ The essential role of BAT in systemic thermal homeostasis has been well established in rodents. For example, mice lacking functional BAT rapidly succumb to acute 4°C cold stress.^3^^,^^4^^,^^5^

In brown adipocytes, sympathetic stimulation activates cAMP signaling to drive mitochondrial biogenesis and uncoupling protein 1 (Ucp1)-mediated proton leak, both of which are required for non-shivering thermogenesis.^1^^,^^3^ Disruption of this pathway, as observed in the betaless mice (null for all three β-adrenergic receptors [βARs]) or in brown-adipocyte-specific adenylate-cyclase-stimulating Gα-deficient mice, leads to reduced expression of the mitochondrial biogenesis regulator PGC1α, loss of multilocular morphology (“BAT whitening”), impaired β-adrenergic oxygen consumption, and heightened cold sensitivity. Similar defects are observed in *Ucp1*-knockout mice, underscoring the critical importance of mitochondrial quantity and Ucp1-dependent uncoupling in brown fat thermogenesis.

Mitochondrial respiration is powered by the electron transport chain (ETC), which establishes the proton motive force used by Ucp1 to dissipate energy as heat. We previously demonstrated that disrupting mitochondrial gene expression by deleting mitochondrial transcription factor A (Tfam) or leucine-rich PPR motif-containing protein (Lrpprc) in brown adipocytes abolishes the multilocular morphology, mitochondrial respiration, and Ucp1-dependent thermogenesis, but it induces an alternative thermogenic mechanism that is fueled by ATF4-dependent proteome turnover instead.^6^^,^^7^ Importantly, this alternative ATF4-dependent thermogenesis can replace Ucp1-driven mitochondrial thermogenesis. In contrast, this alternative ATF4-dependent thermogenesis is not observed in mice with reduced mitochondrial biogenesis (e.g., brown-adipocyte-specific *Gnas*-knockout and betaless mice), which lack cold-induced increases in energy expenditure.^8^^,^^9^^,^^10^

Cytochrome *c* oxidase (complex IV), the last and non-redundant component of ETC, is essential for establishing the proton gradient. Its biogenesis depends on the heme A-synthesis factor Cox10, which is indispensable for complex IV assembly.^11^ Loss of Tfam or Lrpprc reduces complex IV activity after ∼90% reduction of mtDNA-encoded gene expression. Here, we investigate the consequences of directly impairing complex IV function through brown-adipocyte-specific deletion of *Cox10*, a gene required for complex IV assembly. In Cox10^BKO^ mice, severe complex IV deficiency abolished mitochondrial respiration and Ucp1-dependent thermogenesis. Although ATF4 signaling was induced in Cox10-deficient brown adipocytes, the alternative ATF4-dependent thermogenic program failed to engage due to a blockade of protein synthesis. Unexpectedly, Cox10^BKO^ mice were resistant to high-fat diet (HFD)-induced obesity and insulin resistance despite defective BAT thermogenesis. Future studies are warranted to elucidate the mechanisms through which respiration-deficient brown adipocytes promote metabolic fitness in mice.

## Results

### Cox10 is essential for mitochondrial respiration in brown adipocytes

**Figure 1:**
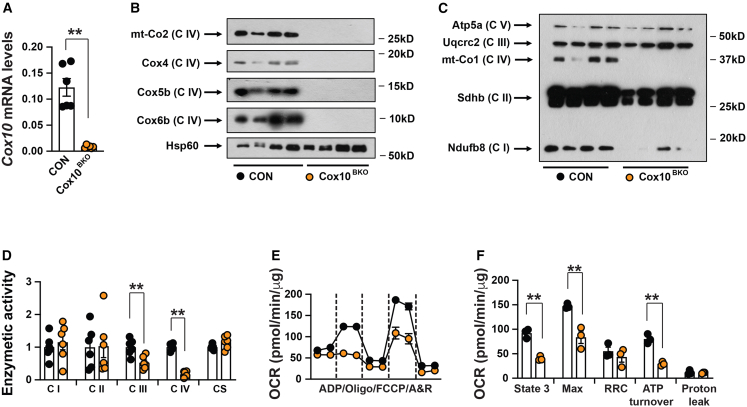
Cox10 is essential for mitochondrial respiration in brown adipocytes (A) qPCR of *Cox10* mRNA levels in the iBAT of ∼8-week-old male CON (genotype: *Cox10*^*f/f*^) and Cox10^BKO^ (genotype: *Ucp1-Cre*;*Cox10*^*f/f*^) mice. *n* = 6 mice per group. Data are presented as mean ± SEM. (B) Immunoblots of complex IV subunits (mt-Co2, Cox4, Cox5b, and Cox6b) and Hsp60 in isolated mitochondria from the aforementioned mice. Representative blots from at least three independent experiments. (C) Immunoblots of mitochondrial proteins from 5 respiration complexes (Ndufb5 for C I, Sdhb for C II, Uqcrc2 for C III, mt-Co1 for C IV, and Atp5a for C V) in isolated mitochondria from the aforementioned mice. Representative blots from at least three independent experiments. (D) Relative *in vitro* enzyme activities of complexes I to IV and citrate synthase (CS) in the BAT of ∼8-week-old male CON and Cox10^BKO^ mice. *n* = 6 mice per group. Data are presented as mean ± SEM. Statistical significance was determined by two-tailed Student’s *t* test. (E) Seahorse experiments measuring oxygen consumption rates (OCRs) of isolated mitochondria from the BAT of ∼8-week-old male CON and Cox10^BKO^ mice upon addition of ADP, Oligo, FCCP, and A&R. Representative traces from three independent experiments. *n* = 3 mice per group. Data are presented as mean ± SEM. Statistical significance was determined by two-tailed Student’s *t* test. (F) State 3, ATP turnover, and maximum OCRs in Seahorse measurements. Values are normalized as per mitochondrial protein. Representative traces from three independent experiments. *n* = 3 mice per group. Data are presented as mean ± SEM. Statistical significance was determined by two-tailed Student’s *t* test. ∗*p* < 0.05 and ∗∗*p* < 0.01.

Cox10 is an indispensable factor in complex IV assembly, and its loss leads to complex IV deficiency.^12^ To determine the specific role of Cox10 in brown adipocytes, we generated brown-adipocyte-specific Cox10-knockout mice (*Ucp1-Cre*;*Cox10*^*f/f*^, hereinafter referred to as Cox10^BKO^). qPCR analysis confirmed efficient deletion of *Cox10* transcript in the interscapular BAT (iBAT) of Cox10^BKO^ mice (Figure 1A). As we expected, steady-state protein levels of complex IV subunits (mtDNA-encoded mt-Co1 and mt-Co2, and nuclear-encoded Cox4, Cox5b, and Cox6b) were reduced in the isolated mitochondria from the iBAT of Cox10^BKO^ mice (Figure 1B). Among other ETC components, Ndufb8 (from complex I) was also reduced, whereas representative subunits from complexes II (Sdhb), III (Uqcrc2), and V (Atp5a) remained unchanged (Figure 1C). In contrast, mRNA levels for both mitochondria-encoded and nuclear-encoded ETC genes were not significantly affected in the BAT of the Cox10^BKO^ mice (Figures S1A and S1B), indicating intact transcription despite loss of protein components.

Consistently, complex IV enzyme activity *in vitro* was significantly attenuated in the iBAT of Cox10^BKO^ mice (∼20% remaining) (Figure 1D). Complex III activity was also reduced by approximately 50% (Figure 1D), likely reflecting secondary instability within the ETC. Measurements of mitochondrial function using Seahorse assays on isolated mitochondria further demonstrated profound energetic defects in Cox10-deficient mice, including reductions in state 3 respiration, maximum respiration, and oligomycin-sensitive ATP production (ATP turnover). In contrast, both the spare respiration capacity (RRC) and proton leak were not affected (Figures 1E and 1F). Collectively, the Cox10^BKO^ mice, like the brown-adipocyte-specific Tfam- and Lrpprc-knockout mice we have previously characterized,^6^^,^^7^ exhibit profound impairments in mitochondrial respiratory capacity specifically within BAT.

### The Cox10^BKO^ mice are defective in cold-induced thermogenesis

**Figure 2:**
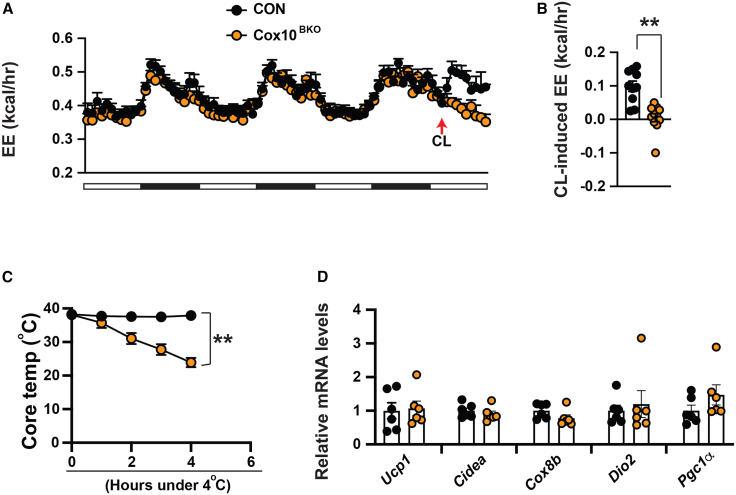
The Cox10^BKO^ mice are defective in cold-induced thermogenesis (A) Recordings of energy expenditure (EE, kcal/h) in ∼8-week-old male CON and Cox10^BKO^ mice for 3 days. Red arrowhead indicates time of CL 316,423 (CL) injection. *n* = 10 control and *n* = 11 Cox10^BKO^ mice. (B) Average hourly CL-induced EE in the aforementioned experiment. *n* = 10 control and *n* = 11 Cox10^BKO^ mice. Data are presented as mean ± SEM. Statistical significance was determined by Student’s *t* test. (C) Cold tolerance test (CTT) of ∼8-week-old male CON and Cox10^BKO^ mice. *n* = 5 control and *n* = 7 Cox10^BKO^ mice. Data are presented as mean ± SEM. (D) qPCR analysis of thermogenic genes (*Ucp1*, *Cidea*, *Cox8b*, *Dio2*, and *Pgc1a*) in the iBAT of ∼8-week-old male CON and Cox10^BKO^ mice. *n* = 6 mice per group. Data are presented as mean ± SEM. Student’s *t* test. ∗*p* < 0.05, ∗∗*p* < 0. 01.

Since mitochondrial respiration fuels βAR-induced adaptive thermogenesis in BAT through Ucp1-mediated uncoupling,^1^ we next assessed whole-body energy metabolism in 8–10 weeks old male mice Cox10^BKO^ and control mice using indirect calorimetry experiments. At the basal state, Cox10^BKO^ mice displayed normal energy expenditure (EE), respiratory exchange ratio (RER), food intake, and physical activity compared with controls (Figures S2A–S2D), indicating that Cox10 deletion in brown adipocytes does not perturb resting metabolic homeostasis. However, β3 agonist CL 316,423 (CL) stimulation significantly induced EE in control mice, whereas Cox10^BKO^ mice failed to induce any thermogenic response (Figures 2A and 2B). Consistent with this defect, Cox10^BKO^ mice exhibited profound cold intolerance: during a 4°C cold-challenge test, they rapidly lost core body temperature at a rate comparable to Ucp1-knockout mice (Figure 2C). Notably, expression of key thermogenic genes, such as *Ucp1*, *Cidea*, *Cox8b*, *Dio2*, and *Pgc1α*, was unaffected by Cox10 deficiency (Figure 2D), indicating that the thermogenic defect arises from impaired mitochondrial function rather than transcriptional downregulation of the thermogenic program. Collectively, these results establish Cox10 as essential for maintaining complex-IV-dependent mitochondrial respiration in brown adipocytes and β-adrenergic thermogenic capacity in iBAT and systemic thermoregulation.

### Cox10 deficiency activates the ATF4 transcription program but fails to induce ATF4-dependent thermogenesis in brown adipocytes

We previously reported that loss of mtDNA-gene expression in brown adipocytes (in Lrpprc^BKO^ and Tfam^BKO^ mice) induces an ATF4-driven transcriptional program, enhances cytosolic proteome turnover, and supports a Ucp1-independent thermogenic process.^6^^,^^7^ To determine whether Cox10 deficiency elicits a similar response, we examined ATF4 pathway activation in Cox10-deficient brown adipocytes.

**Figure 3:**
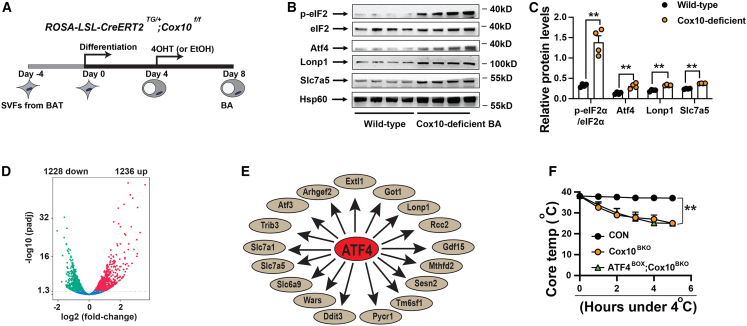
Cox10 deficiency activates the ATF4 transcription program in brown adipocytes (A) Strategy to generate Cox10-deficient brown adipocytes. (B) Immunoblots showing amounts of p-eIF2α, total eIF2α, Atf4, Lonp1, Scl7a5, and Hsp90 in wild-type and Cox10-deficient brown adipocytes. Representative blots from at least three independent experiments. (C) Quantifications of the ratio of p-eIF2α to total eIF2α, Atf4, Lonp1, and Scl7a5 levels in wild-type and Cox10-deficient brown adipocytes. *n* = 4 independent experiments. Data are presented as mean ± SEM. (D) Volcano plots showing significantly (*p* < 0.05) down- or up-regulated genes in the BAT of Cox10^BKO^ mice. *n* = 3 biological replicates per group. (E) ATF4 transcription program (up-regulated genes in the Cox10^BKO^ mice shown). (F) CTT of ∼8-week-old male CON, Cox10^BKO^, and Cox10^BKO^;ATF4^BOX^ mice. *n* = 11 control, *n* = 3 Cox10^BKO^, and *n* = 4 Cox10^BKO^;ATF4^BOX^ mice. Data are presented as mean ± SEM. Student’s *t* test. ∗*p* < 0.05, ∗∗*p* < 0.01.

To model Cox10 loss *in vitro*, we isolated stromal vascular fractions (SVFs) from the iBAT of ROSA-LSL-CreERT2ˆTG/+;Cox10f/f pups (7–10 days old), differentiated them into mature brown adipocytes, and induced Cox10 deletion post-differentiation by treating cells with 4-hydroxytamoxifen (4OHT) (Figure 3A). CreERT2-mediated recombination effectively deleted Cox10, resulting in robust phosphorylation of eIF2α—an upstream event in the integrated stress response (ISR) and a surrogate marker of ATF4 activation. Correspondingly, ATF4 protein levels and the expression of canonical ATF4 target genes such as *Lonp1* and *Slc7a5* were markedly increased (Figures 3B and 3C).

To assess transcriptional remodeling *in vivo*, we performed RNA-seq experiments on the iBAT of Cox10^BKO^ mice fed normal chow and compared the differentially expressed genes (DEGs). The ETC genes were not altered, consistent with the result from qPCR analysis (Figures 5 and S1). The volcano plot showed that over 1,200 genes were significantly up- and down-regulated in the iBAT of Cox10^BKO^ mice with a >2-fold difference (Figure 3D). Among the upregulated genes were numerous established ATF4 targets, confirming activation of the ATF4 stress-response program in Cox10-deficient brown adipocytes (Figure 3E).

We previously demonstrated that ATF4 overexpression in wild-type brown adipocytes is sufficient to initiate ATF4-dependent thermogenesis and even improve cold tolerance in Ucp1-knockout mice. In contrast, ATF4 overexpression in the brown adipocytes of Cox10^BKO^ mice (Ucp1-Cre;ROSA-LSL-ATF4;Cox10f/f, ATF4^BOX^;Cox10^BKO^) failed to increase the cold resistance upon a 4°C cold tolerance test (Figure 3F). Therefore, ATF4 activation is sufficient to drive the ATF4-dependent thermogenesis in wild-type or Ucp1-deficient, Lrpprc- or Tfam-deficient brown adipocytes, but not in Cox10-deficient brown adipocytes.

### Ribosome biogenesis is required for ATF4-dependent protein turnover in adipocytes

**Figure 4:**
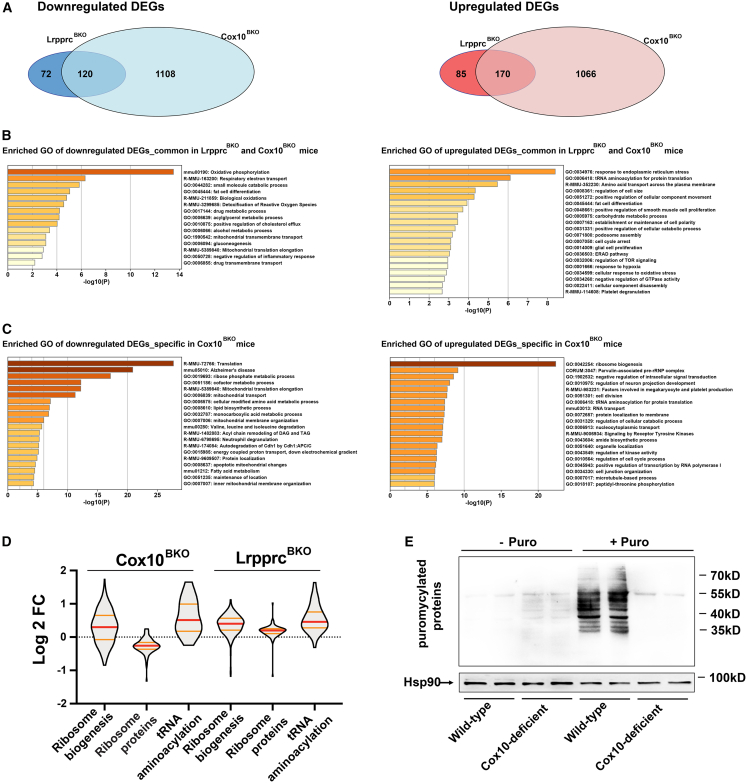
Ribosome biogenesis is required for ATF4-dependent protein turnover in brown adipocytes (A) Venn diagrams showing the numbers of down- and up-regulated DEGs in the iBAT of Cox10^BKO^ and Lrpprc^BKO^ mice. (B) Most enriched GO terms in down- and up-regulated DEGs shared in Cox10^BKO^ and Lrpprc^BKO^ mice. (C) Most enriched GO terms in down- and up-regulated DEGs unique to Cox10^BKO^ mice. (D) Violin plot showing the log2 fold-change of genes involved in ribosome biogenesis, ribosome proteins, and tRNA aminoacylation in the iBAT of Cox10^BKO^ and Lrpprc^BKO^ mice. *n* = 3 biological replicates per group. (E) Immunoblots of puromycylated protein and Hsp90 in wild-type and Cox10-deficient brown adipocytes Representative images from at least two independent experiments.

To better understand why Cox10-deficient brown adipocytes fail to engage ATF4-dependent thermogenesis, we compared the DEGs in iBAT from Lrpprc^BKO^ mice (GSE117985) and Cox10^BKO^ mice. As expected, the most significantly enriched Gene Ontology (GO) categories for down-regulated and up-regulated DEGs shared in both models included mitochondrial oxidative respiration and ATF4-associated cytosolic processes, such as endoplasmic reticulum (ER) stress responses and protein translation (Figures 4A and 4B). In fact, the increased ATF4 gene signature, represented as a typical response to defective mitochondrial function, was also observed in the heart (GSE96518)^13^ (Figure S4B). Notably, however, Cox10^BKO^ mice displayed a distinct transcriptomic signature, with genes involved in cytosolic protein translation (R_MMU-72766) being specifically downregulated (Figure 4A). A more detailed analysis revealed that transcripts encoding ribosomal proteins were selectively reduced in the iBAT of Cox10^BKO^ mice, whereas genes involved in ribosome biogenesis or tRNA aminoacylation remained unaffected. The decrease in ribosomal protein gene expression was not observed in the iBAT of Lrpprc^BKO^ mice or in the heart with defective ETC gene expression (Figures 4D and S4C), suggesting that repression of ribosomal protein gene expression is a unique feature caused by Cox10 deficiency.

Consistent with these transcriptomics changes, Cox10-deficient brown adipocytes exhibited a severe inhibition of global protein synthesis, as shown by markedly reduced incorporation of puromycin into nascent polypeptides (Figure 4E). This impairment of protein synthesis seemed to be mTORC1-independent, because phosphorylation of 4EBP1 and S6 remained unchanged in the Cox10-deficient brown adipocytes (Figures S4A and S4B). Together, these findings suggest that Cox10 loss impairs the expression of ribosomal proteins and global protein synthesis in brown adipocytes. As a result, the ATF4-driven thermogenic program, which relies on a high-flux cycle of protein synthesis and degradation, cannot be engaged in Cox10^BKO^ mice due to insufficient translational capacity.

### Cox10^BKO^ mice exhibit improved systemic metabolism under high-fat diet feeding

**Figure 5:**
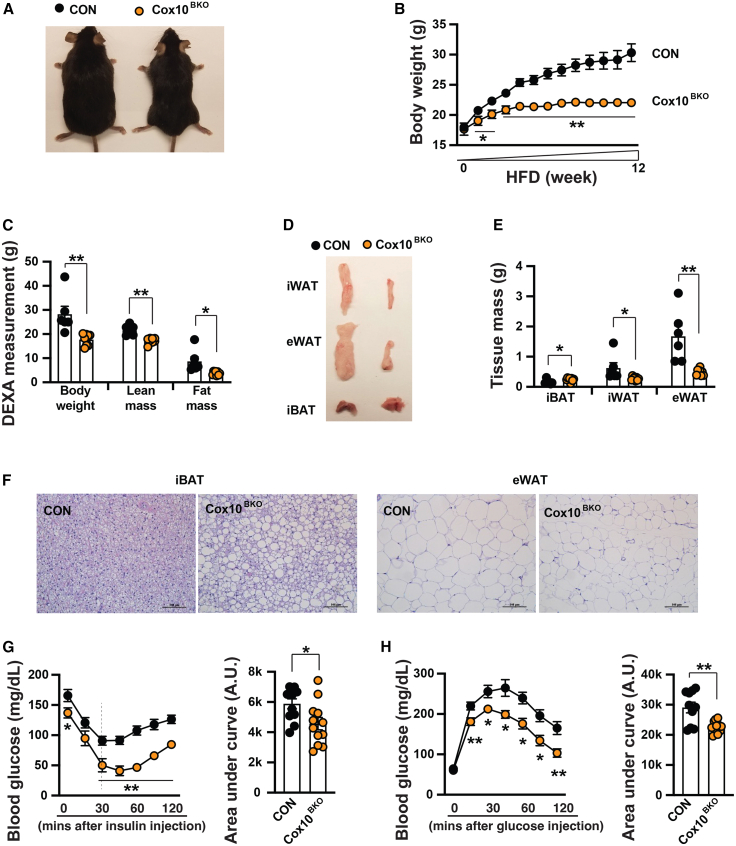
Cox10^BKO^ mice exhibit improved systemic metabolism under high-fat diet feeding (A) Representative images of male CON and Cox10^BKO^ mice under 12 weeks of high-fat diet (HFD). (B) Body weight of male CON and Cox10^BKO^ mice under 12 weeks of HFD. *n* = 11 control and *n* = 13 Cox10^BKO^ mice. Data are presented as mean ± SEM. (C) Body weight, lean mass, and fat mass of male CON and Cox10^BKO^ mice after HFD. *n* = 6 control and *n* = 8 Cox10^BKO^ mice. (D) Representative images of dissected iWAT, eWAT, and iBAT from male CON and Cox10^BKO^ mice after HFD. (E) Tissue mass of eWAT, iWAT, and iBAT of male CON and Cox10^BKO^ mice after HFD. *n* = 6 control and *n* = 8 Cox10^BKO^ mice. Data are presented as mean ± SEM. (F) Representative H&E staining of iBAT and eWAT from male CON and Cox10^BKO^ mice after HFD. Images representative of at least two independent experiments. Scale bars, 100 μm. (G) Left: serum glucose levels during ITT in male CON and Cox10^BKO^ mice after HFD. Right: area under the curve (AUC) values of glucose levels in first 30 min of ITTs shown. *n* = 11 control and *n* = 13 Cox10^BKO^ mice. Data are presented as mean ± SEM. (H) Left: serum glucose levels during GTT in male CON and Cox10^BKO^ mice after HFD. Right: area under the curve (AUC) values of glucose levels in GTTs shown. ∗*n* = 10 control and *n* = 11 Cox10^BKO^ mice. Data are presented as mean ± SEM. Student’s *t* test. ∗*p* < 0.05, ∗∗*p* < 0.01.

To investigate the impact of impaired brown adipocyte protein synthesis on systemic metabolism, we determined metabolic performance in the Cox10^BKO^ mice subjected to an HFD. Male Cox10^BKO^ mice showed a leaner phenotype compared with control (CON) mice after feeding HFD for ∼12 weeks (Figures 5A and 5B). Despite starting with similar body weights, Cox10^BKO^ mice gained significantly less weight over the HFD period (Figure 5B). Correspondingly, total adiposity was reduced, as evidenced by smaller fat depots, including inguinal white adipose tissue (iWAT), epididymal white adipose tissue (eWAT), and interscapular brown adipose tissue (iBAT) (Figures 5C–5E). Histological analysis revealed whitening of brown adipocytes in the iBAT and reduced white adipocyte size in the eWAT (Figure 5F), indicating that reduced fat accumulation contributes to the lower body weight of Cox10^BKO^ mice under HFD conditions.

Other HFD-induced metabolic parameters, such as glucose homeostasis and insulin sensitivity, were also measured in male Cox10^BKO^ mice. As observed by glucose tolerance test (GTT) and insulin tolerance test (ITT) on HFD-fed mice, basal blood glucose levels were significantly lower in Cox10^BKO^ mice compared with CON mice (Figures 5G and 5H). These improvements in HFD-induced obesity and insulin resistance were also observed in the female Cox10^BKO^ mice (Figure S5). Hence, these results demonstrate that Cox10 deficiency in brown adipocytes confers resistance to diet-induced obesity and metabolic dysfunction, despite a severe impairment of adaptive thermogenesis.

WAT browning, the formation of Ucp1+multilocular beige adipocytes within white adipose tissues, is associated with metabolic improvements such as reduced adiposity and improved glycemic control. We found that the Cox10^BKO^ mice did exhibit WAT browning, showing elevated thermogenic gene (*Ucp1*, *Cidea*, and *Cox8b*) expression and the presence of the multilocular beige adipocytes in the iWAT of the Cox10^BKO^ mice even after HFD (Figures S6A and S6B). Notably, this phenotype was also evident in the Tfam^BKO^ and Lrpprc^BKO^ mice.^7^

### The metabolic benefits in the Cox10^BKO^ mice persist at thermoneutrality

**Figure 6:**
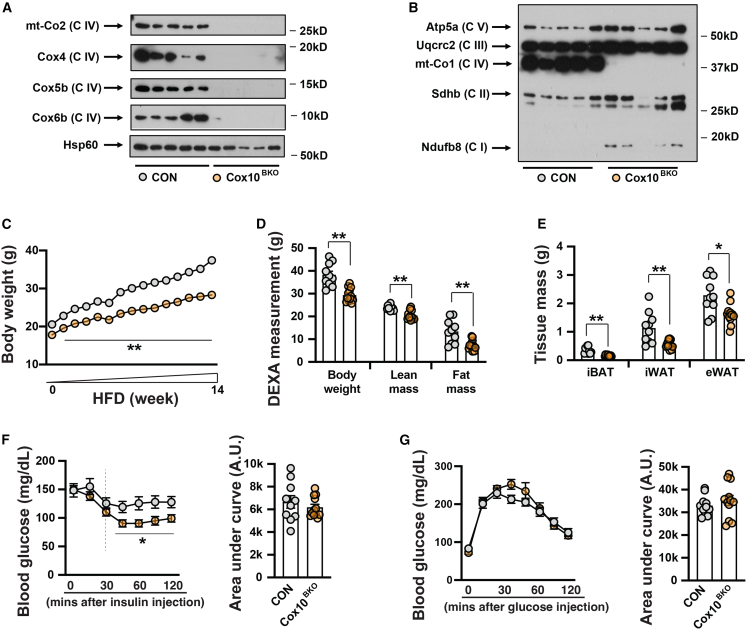
Phenotypes of the Cox10^BKO^ mice persist at thermoneutral housing (A) Immunoblots of complex IV subunits (mt-Co2, Cox4, Cox5b, and Cox6b) and Hsp60 in isolated mitochondria of iBAT from male CON and Cox10^BKO^ mice housed at 30°C. (B) Immunoblots of mitochondrial proteins from 5 respiration complexes (Ndufb5 for C I, Sdhb for C II, Uqcrc2 for C III, mt-Co1 for C IV, and Atp5a for C V) in isolated mitochondria from the aforementioned mice. Representative images from at least three independent experiments. (C) Body weight of male CON and Cox10^BKO^ mice under 14 weeks of HFD at 30°C. *n* = 12 mice per group. (D) Body weight, lean mass, and fat mass of male CON and Cox10^BKO^ mice after HFD at 30°C. Sample size: CON (*n* = 10) and Cox10^BKO^ (*n* = 12). (E) Tissue mass of eWAT, iWAT, and iBAT of male CON and Cox10^BKO^ mice after HFD at 30°C. Sample size: CON (*n* = 10) and Cox10^BKO^ (*n* = 12). (F) Left: serum glucose levels during ITT in male CON and Cox10^BKO^ mice after HFDat 30°C. Right: area under the curve (AUC) values of glucose levels in first 30 min of ITTs shown. Sample size: CON (*n* = 11) and Cox10^BKO^ (*n* = 13). (G) Left: serum glucose levels during GTT in male CON and Cox10^BKO^ mice after HFD at 30°C. Right: area under the curve (AUC) values of glucose levels in GTTs shown. Sample size: CON (*n* = 10) and Cox10^BKO^ (*n* = 11). Data are presented as average ±SEM. Student’s *t* test. ∗*p* < 0.05 and ∗∗*p* < 0.01.

The Ucp1-knockout mice are cold intolerant and are only obesity-prone after HFD feeding at thermoneutrality.^14^ To determine whether the metabolic benefits in the Cox10^BKO^ mice at room temperature (RT) stemmed from secondary adaptive responses from defective thermoregulation, we housed newly weaned Cox10^BKO^ mice at 30°C for 4 weeks and we analyzed the ETC protein expression in the isolated mitochondria of the iBAT. Similar to the results observed at RT, the steady-state levels of complex IV ETC proteins were reduced without changes of their transcript levels (Figure 6A-B, S7A, and S7B). The Cox10^BKO^ mice still failed to respond to CL-induced energy expenditure (Figures S8A and S8B), without significant changes in EE and RER (Figures S8C and S8D). Total activity and food intake were reduced (Figures S8E and S8F). We then performed HFD feeding experiments on the male CON and Cox10^BKO^ mice at 30°C for additional 14 weeks. The Cox10^BKO^ mice were still protected against HFD-induced obesity (Figures 6C–6E). However, the Cox10^BKO^ mice were no longer glucose intolerant; they were more insulin sensitive in the ITT assay due to an enhanced counter regulatory response (Figures 6F and 6G). Together, these findings further suggest that brown adipocytes can modulate systemic metabolic homeostasis through mechanisms independent of Ucp1-mediated thermogenesis.

## Discussion

Humans maintain relatively stable body weight through precise regulation of energy balance, which requires a tight coupling between energy intake from food and energy expenditure (EE) for basal metabolism (to sustain organ function), physical activity, and adaptive thermogenesis (to generate heat and maintain core body temperature When energy intake chronically exceeds expenditure, the surplus is stored as fat, leading to increased adiposity and associated metabolic disturbances. While human genome-wide association study (GWAS) studies suggest that hyperphagia (increased food intake) may be a primary driver of obesity, the concept of diet-induced thermogenesis (DIT), or energy dissipation in response to nutrient intake, termed initially “Luxuskonsumptionˮ by Neumann in 1902, highlights the role of energy expenditure in maintaining metabolic homeostasis. BAT has long been proposed as an effector of DIT, offsetting the positive energy balance since 1979, although this idea remains debated.^15^^,^^16^

We have previously unexpectedly identified an ATF4-dependent thermogenic program in brown adipocytes that operates independently of Ucp1 and can adjust energy expenditure according to diet composition.^7^ Physiological evidence for Ucp1-independent thermogenesis is also observed in young pigs, characterized by a lack of Ucp1 expression,^17^^,^^18^ and in humans with complete spinal cord injury, who have impaired sympatho-adrenal activity.^19^ These observations underscore the potential relevance of alternative thermogenic mechanisms and suggest the existence of a previously unrecognized thermoregulatory axis that warrants further investigation.

In models such as the Tfam^BKO^ and Lrpprc^BKO^ mice, respiration defects in brown adipocytes lead to the coordinated activation of the ATF4 and mTORC1 pathways, promoting protein synthesis.^7^ ATF4 transcriptionally upregulates aminoacyl-tRNA synthetases and amino acid transporters (AATs), which are required for translation elongation. AAT-mediated amino acid uptake, in turn, activates mTORC1-dependent protein synthesis, particularly under stress conditions.^20^^,^^21^^,^^22^ However, mTORC1 can also reciprocally regulate ATF4 mRNA stability and translation.^23^

However, despite exhibiting similar mitochondrial respiration defects and ATF4 activation as the Tfam^BKO^ and Lrpprc^BKO^ mice, the Cox10^BKO^ mice fail to engage the Ucp1-independent thermogenesis in the iBAT. Comparative transcriptomic analysis of the iBAT from Lrpprc^BKO^ and Cox10^BKO^ mice revealed that Cox10-deficient brown adipocytes specifically downregulated ribosomal protein genes, likely contributing to impaired global protein synthesis. Interestingly, while Cox10-deficient natural killer cells exhibited increased mTORC1 activity during viral infection,^24^ we observed comparable mTORC1 phosphorylation levels in Cox10-deficient mouse brown adipocytes, suggesting that the modulation of the mTORC1 activity by Cox10-deficiency is context-dependent. The mechanistic basis for the divergent outcomes of respiration-defective mitochondria in Lrpprc-versus Cox10-deficient brown adipocytes remains unknown, particularly regarding cytosolic protein synthesis.

Importantly, although mitochondrial ATP turnover is significantly reduced in Cox10-deficient brown adipocytes (Figure 1F), this measurement reflects ATP production capacity rather than steady-state cellular ATP content. Brown adipocytes are intrinsically adapted for low coupling efficiency due to Ucp1-mediated proton leak, and oxidative phosphorylation in this tissue is primarily dedicated to heat production rather than maximal ATP generation.^1^ Therefore, reduced mitochondrial ATP turnover does not necessarily imply depletion of total cellular ATP pools.^25^ Compensatory mechanisms, such as increased glycolytic flux and cytosolic substrate-level phosphorylation, may help maintain ATP homeostasis despite impaired complex IV function. This distinction may explain why Cox10-deficient brown adipocytes exhibit impaired translational capacity associated with ribosomal gene downregulation, rather than a simple ATP-limitation-driven suppression of protein synthesis. Direct quantification of total ATP content *in vivo* will be required to fully resolve this question.^26^^,^^27^

Our results further refine our understanding of ATF4 signaling in adipocytes. While ATF4 is essential for cold-induced thermogenesis in Ucp1-expressing or Tfam/Lrpprc-deficient models, loss of translational capacity uncouples ATF4 activation from thermogenic outcomes. Moreover, recent studies indicate that ATF4 can exert context-dependent metabolic effects: adipocyte-specific ATF4 deletion improves insulin sensitivity under HFD, likely via modulation of BAT inflammation, without affecting body weight.^28^ Thus, the metabolic consequences of ATF4 activation are determined by the interplay between ISR engagement, translational capacity, and mitochondrial integrity.

Beyond cell-autonomous thermogenic regulation, our data reveal that respiration-deficient brown adipocytes confer systemic metabolic benefits. Cox10^BKO^ mice exhibit reduced adiposity, improved glucose tolerance, and enhanced insulin sensitivity under HFD despite defective BAT thermogenesis. This aligns with emerging evidence that mitochondrial stress induces endocrine signals, or mitokines, that remodel whole-body metabolism. In models of OPA1 or PDSS2 deficiency in BAT, ATF4-driven secretion of FGF21 and GDF15 mediates resistance to diet-induced obesity and supports energy homeostasis, even in the absence of Ucp1 activity.^29^^,^^30^^,^^31^ Similarly, FGF21 and GDF15 are central effectors of the ISR in multiple tissues, acting as endocrine mediators to improve metabolic resilience. Our findings suggest that Cox10^BKO^ adipocytes may similarly engage mitokine signaling to promote systemic metabolic fitness independently of heat production.

In conclusion, our findings suggest respiration-deficient brown adipocytes can improve systemic metabolism through a combination of ATF4 activation, translational regulation, and likely mitokine signaling. Looking forward, several avenues merit further investigation. First, defining the mitokine repertoire in Cox10^BKO^ mice, including FGF21, GDF15, and potentially novel BAT-derived factors, is critical to dissect endocrine versus local paracrine contributions to metabolic protection. For example, mitokine Gdf15 may reduce food intake in the Lrpprc^BKO^ mice (maybe Cox10^BKO^) mice and protect HFD-induced obesity at thermoneutrality (Figures S4B and S8E).^7^ Second, elucidating the mechanisms linking complex IV deficiency to ribosomal protein downregulation may reveal new regulatory nodes connecting mitochondrial dysfunction to cytosolic protein synthesis. Finally, restoring translational capacity in Cox10-deficient adipocytes could test the causal role of proteome turnover in ATF4-dependent thermogenesis and clarify whether the release of mitokines alone is sufficient to confer systemic metabolic benefits.

### Limitations of the study

Although our findings reveal that Cox10 deficiency uncouples ATF4 activation from thermogenesis and improves systemic metabolism, several limitations remain. First, circulating mitokines, such as FGF21 and GDF15, were not directly measured, leaving the endocrine mechanisms underlying systemic metabolic benefits unresolved. Second, ribosomal protein downregulation was observed at the transcript level, but mechanistic links between complex IV deficiency and translational suppression remain to be determined. Third, ATP levels were not directly quantified *in vivo*, which would clarify whether energetic stress contributes to impaired protein synthesis. Finally, rescue experiments restoring translational capacity were not performed and would help establish causality between impaired protein synthesis and defective ATF4 dependent thermogenesis.

## Resource availability

### Lead contact

Requests for further information and resources should be directed to and will be fulfilled by the lead contact, Biao Wang (biao.wang@ucsf.edu).

### Materials availability

This study did not generate new, unique reagents.

### Data and code availability

• RNA-seq datasets generated in this study have been deposited in the Gene Expression Omnibus (GEO) under accession number GSE316308. Reviewer access is available using the provided token and will be made publicly accessible upon publication.
• This paper does not report original code.
• Any additional information required to reanalyze the data reported in this paper is available from the lead contact upon request.

## Acknowledgments

This work is supported by 10.13039/100000002National Institutes of Health (NIH) grant nos. DK128459 and DK134490 to B.W. E.P. is supported by a fellowship grant from 10.13039/100001167Hillblom Foundation. The authors would like to thank Christophe Paillart and Vassily Kutyavin for assistance with indirect calorimetry experiments.

## Author contributions

Conceptualization, B.W. and E.P.; methodology, E.P., Y.W., and B.W.; investigation, E.P., Y.W., and B.W; writing – original draft, E.P. and B.W.; writing – review and editing, E.P., Y.W., and B.W.; funding acquisition, B.W.; resources, B.W.; supervision, B.W.

## Declaration of interests

The authors declare no competing interests.

## Declaration of generative AI and AI-assisted technologies in the writing process

The authors declare that no generative AI or AI-assisted technologies were used in the writing process of this manuscript.

## STAR★Methods

### Key resources table

**Table:** 

REAGENT OR RESOURCE	SOURCE	IDENTIFIER
**Antibodies**		

Ucp1 antibody	Sigma	Cat# U6382
Atf4 antibody	Cell Signaling Technology	Cat# 11815
Phospho-eIF2α antibody	Cell Signaling Technology	Cat# 3398
eIF2α antibody	Cell Signaling Technology	Cat# 5324
Phospho-S6 antibody	Cell Signaling Technology	Cat# 5364
S6 antibody	Cell Signaling Technology	Cat# 2217
Phospho-4EBP1 antibody	Cell Signaling Technology	Cat# 2855
4EBP1 antibody	Cell Signaling Technology	Cat# 9452
Lonp1 antibody	Proteintech	Cat# 66043-1-Ig
Slc7a5 antibody	Proteintech	Cat# 13752-1-AP
Hsp90 antibody	Santa Cruz Biotechnology	Cat# SC-7949
Total OXPHOS antibody cocktail	Abcam	Cat# ab110413; RRID: AB_2629281
mt-Co2 antibody	Proteintech	Cat# 55070-1-AP; RRID: AB_10859832
Cox4 antibody	Cell Signaling Technology	Cat# 4850
Cox5b antibody	Bethyl	Cat# A-305-523A
Cox6b antibody	Abgent	Cat# AP20624a
Hsp60 antibody	Bethyl	Cat# A302-846A; RRID: AB_10634219
Puromycin antibody	Kerafast	Cat# EQ0001

**Biological samples**		

Mouse brown adipose tissue	This study	N/A

**Chemicals, peptides, and recombinant proteins**		

Oligomycin A	Sigma	Cat#75351
Sodium pyruvate	Sigma	Cat#SA636
FCCP	Sigma	Cat#C2920
Antimycin A from *Streptomyces* sp.	Sigma	Cat#A8674
Rotenone	Sigma	Cat#R8875
B-NADH	Sigma	Cat#N7410
DCIP	Sigma	Cat#D1878
DTNB	Sigma	Cat#D8130
Acetyl Coenzyme A	Sigma	Cat#A2056
Cytochrome *c*	Sigma	Cat#C3131
Decylubiquinone	Sigma	Cat#D7911
Potassium Borohydride	Sigma	Cat#P4129
Potassium Cyanide	Sigma	Cat#20781
Potassium Ferricyanide	Sigma	Cat#244023
Sodium Hydrosulfite	Sigma	Cat#157953
Insulin solution human	Sigma	Cat#I9278-5 ML
Forskolin	Sigma	Cat#F6886-10 MG
CL316243 disodium salt	Tocris bioscience	Cat#1499
Bio-Rad Protein Assay Solution	BioRad	Cat#500-0006
TRIsure	Bioline	Cat#BIO-38033

**Critical commercial assays**		

XFe24 Flux Assay Kit	Agilent Technologies	Cat#102340-100
Infinity Triglycerides Reagent	Thermo Scientific	Cat#TR22421
QIAamp DNA Mini Kit	Qiagen	Cat#51304
ISOLATE II RNA Mini Kit	Bioline	Cat#BIO-52073
iScript cDNA Synthesis Kit	BioRad	Cat#170-8891
Mouse insulin ultrasensitive ELISA kit	Alpco	Cat#80-INSMSV-E01
Mouse leptin ELISA kit	Crystal Chem	Cat#90030

**Deposited data**		

RNA-seq data (iBAT)	GEO database	GSE316308

**Experimental models: Cell lines**		

Primary brown adipocytes	This study	N/A

**Experimental models: Organisms/strains**		

Cox10 floxed mice	Jackson Laboratory	JAX:024697
Ucp1-Cre mice	Jackson Laboratory	JAX:024670
ROSA-LSL-CreERT2 mice	Jackson Laboratory	JAX:008463
ATF4^BOX^ mice	Paulo, E. et al.^9^	N/A

**Oligonucleotides**		

qPCR primers	This study	Available upon request

**Software and algorithms**		

GraphPad Prism	GraphPad Software	Version 7
ImageJ/Fiji	NIH	https://imagej.nih.gov/ij/

### Experimental model and study participant details

#### Mouse models

Cox10f/f (JAX #024697), Ucp1-Cre (JAX #024670) and ROSA-LSL-CreERT2 (JAX #008463) were obtained from the Jackson Laboratory. ATF4^BOX^ mice were characterized before.^6^ Mice were housed in a temperature-controlled environment at 22°C under a 12h light:dark cycle with free access to water and food (PicoLab® Rodent Diet 20, #5053). For thermoneutral experiments, ∼4-week-old mice are placed in a 30°C rodent chamber (Power Scientific RIS52SD) for an additional 3-4 weeks to reach their thermoneutral zone. There were no inclusion/exclusion criteria for mice studies. Mice were in C57BL/6J background. All animal experiments were approved by the UCSF Institutional Animal Care and Use Committee (AN193603) in adherence to US National Institutes of Health guidelines and policies.

### Method details

#### Metabolic studies

About 8-week-old mice were transferred to a 60% fat diet (Research Diets, D12492). Body weight was monitored once a week. EchoMRI was performed following manufacturer’s instructions. For insulin tolerance test (ITT), mice were fasted 4–6 h before intraperitoneal administration of insulin (Humulin; 0.75U kg^−1^). Blood glucose was measured from tail vein at indicated time points with a glucometer (Contour, Bayer). For ITT analysis, only glucose values from 0 to 30 min were used to calculate the area under the curve (AUC) in order to exclude the counter-regulatory rebound phase at later time points. Serum and liver TG contents were measured by Infinity Triglycerides Reagents (Thermo Scientific, #TR22421). Serum insulin levels were measured by commercial ELISA kits (Alpco, #80-INSMSV-E01).

#### Indirect calorimetry measurements

Basal energy expenditure (EE) and CL-induced EE were calculated per mouse.^32^^,^^33^ Investigators were blinded to the mouse genotypes for CLAMS, which was performed by the UCSF Diabetes and Endocrinology Research Center Metabolic Research Unit.

#### Cold tolerance test (CTT)

∼8-week-old mouse was singly housed with free-access to food and water during CTT. The core body temperatures prior to and during 4°C cold exposure (at 1-h interval) were measured using BAT-12 Microprobe Thermometer with probe RET-3 (Physitemp).

#### ETC complex activities

Frozen BAT tissue from about 8-week-old male and female mice was homogenized in 250 μL homogenization buffer (120 mM KCl, 20 mM HEPES, 1 mM EGTA, pH 7.4) by sonication (5 s pulse x 5, 60% power) using a Microson XL2000 Ultrasonic Cell Disruptor (Misonix). Protein was quantified using the Bradford assay and all samples were diluted to a final concentration of 1 μg/μL of protein. The spectrophotometric kinetic assays were performed using a monochromator microplate reader (Tecan M200 Pro). Complex I activity (NADH:ubiquinone oxidoreductase) was determined by measuring oxidation of NADH at 340 nm (using ferricyanide as the electron acceptor) in a reaction mixture of 50 mM potassium phosphate (pH 7.5), 0.2 mM NADH, and 1.7 mM potassium ferricyanide. Complex II activity (Succinate Dehydrogenase) was determined by measuring the reduction of the artificial electron acceptor 2,6-dichlorophenol-indophenol (DCIP) at 600 nm in a reaction mixture of 50 mM potassium phosphate (pH 7.5), 20 mM succinate, 2 μM DCIP, 10 μM rotenone, and 1 mM potassium cyanide. Complex III activity (Ubiquinol:cytochrome *c* oxidoreductase) was determined by measuring the reduction of cytochrome *c* at 550 nm in a reaction mixture of 50 mM potassium phosphate (pH 7.5), 35 μM reduced decylubiquinone, 15 μM cytochrome *c*, 10 μM rotenone, and 1 mM potassium cyanide. Complex IV activity (Cytochrome *c* oxidase) was determined by measuring the oxidation of cytochrome *c* at 550 nm in a reaction mixture of 50 mM potassium phosphate (pH 7.0) and 100 μM reduced cytochrome *c*. Citrate synthase activity was determined by measuring the reduction of 5,5′-dithiobis (2-nitrobenzoic acid) (DTNB) at 412 nm which was coupled to the reduction of acetyl-CoA by citrate synthase in the presence of oxaloacetate. The reaction mixture consisted of 100 mM Tris-HCl (pH 8.0), 100 μM DTNB, 50 μM acetyl-CoA, and 425 μM oxaloacetate. All activities were calculated as nmoles/min/mg protein, normalized to citrate synthase (CS) activity and finally expressed as the percentage of wild-type activity.

#### Mitochondria isolation

Freshly dissected BAT tissue from about 8-week-old male and female mice was homogenized in a Dounce homogenizer with 5 mL ice-cold mitochondria isolation buffer (210 mM Mannitol, 70 mM Sucrose, 1 mM EGTA, 5 mM HEPES pH7.5, 0.5% BSA). The homogenates were filtered through cheesecloth to remove residual particulates and intact mitochondria were isolated by differential centrifugation using a previously described protocol.^34^ The mitochondrial pellet was resuspended in 25 μL of isolation buffer and protein was quantitated using the Bradford assay (BioRad, #500–0006).

#### Histology

Tissues were fixed in 10% formalin and processed and stained at AML Laboratories. Cell size was measured using ImageJ. Adipocyte size distribution was calculated using total adipocyte numbers counted in multiple images.

#### In vitro adipocytes

Murine adipocytes are differentiated from stromal-vascular fractions (SVF) as previously described.^35^^,^^36^ Briefly, BAT SVF was digested in digestion buffer for 45 min: Ham’s F-10 1× Medium (Corning, #10-070-CV), Collagenase 1.5 mg/mL (Sigma, #C6885) and Dispase II 1 mg/mL (Roche, #14549000). Cells were filtered with a sterile cell strainer (70 μM Nylon mesh, Fisher, #22363548) and centrifuged in growth medium (DMEM medium with 10% FBS) at 2000 rpm for 5 min. Cell pellets were plated in a 6-well cell culture plate until 100% confluency. Adipocyte differentiation was induced by adding differentiation medium: DMEM 10% FBS, 20 nM insulin (Sigma, #I9278), 1 nM T3 (Sigma, #T2877), 0.5 mM IBMX (3-Isobutyl-1-methylxanthine, Sigma, #I5879), 0.5 μM Dexamethasone (Sigma, #D8893), 0.125 mM Indomethacin (Sigma, #I7378), 1 μM Rosiglitazone (Sigma, #R2408) for 4 days. After fully differentiation, 4-Hydroxytamoxifen (4OHT, Sigma# H7904) was added to excise floxed alleles. Ethanol was added for negative control. Differentiated adipocytes were harvested for qRT-PCR and western blots after another 4 days. For Puromycin-chasing experiments, 10 μg/mL Puromycin (ThermoFisher, #A1113803) was added for 10 min.

#### Immunoblots

For lysates, tissues were lysed in ice-cold lysis-buffer (50 mM Tris-HCl, 150 mM NaCl, 1 mM EDTA, 6 mM EGTA, 20 mM NaF, 1% Triton X-100, 1 μM MG132 and protease inhibitors) using a TissueLyser II (Qiagen). After centrifugation at 13000 rpm for 15 min, supernatants were reserved for protein determinations and SDS-PAGE analysis. Mitochondria were lyzed in the above lysis buffer before immunoblotting. Antibodies used were: Ucp1 (Sigma, #U6382), Atf4 (Cell Signaling Technology, #11815), *p*-eIF2α (Cell Signaling Technology, #3398), eIF2α (Cell Signaling Technology, #5324), p-S6 (Cell Signaling Technology, #5364), S6 (Cell Signaling Technology, #2217), p-4Ebp1 (Cell Signaling Technology, #2855), 4Ebp1 (Cell Signaling Technology, #9452), Lonp1 (Proteintech, #66043-1-Ig), Slc7a5 (Proteintech, #13752-1-AP), Hsp90 (Santa Cruz Biotechnology, #SC-7949), total OXPHOS protein (Abcam, #ab110413), mt-Co2 (Proteintech, #55070-1-A), Cox4 (Cell Signaling Technology, #4850), Cox5b (Bethyl, #A-305-523A), Cox6b (Abgent, #AP20624a), Hsp60 (Bethyl, #A302-846A), and puromycin (Kerafast, #EQ0001). Quantification of relative protein expression was performed using Fiji ImageJ software.

#### Q-PCR and RNA-seq

IBI Tri-Isolate RNA Pure Kit (IBI Scientific, #76006–526) was used to extract total RNA from tissues and adipocytes. Purified RNA was reversely transcribed into cDNA with PrimeScript™ RT reagent Kit (Takara, #RR037A). cDNA was used for qRT-PCR on a CFX384 real-time PCR detection system (Bio-Rad). Relative mRNA expression levels were calculated using the 1/2^ΔCt^ method with m36B4 as the internal reference control. RNA-seq was performed by Novogene Inc. Briefly, first strand cDNA was synthesized using random hexamer primer and M-MuLV Reverse Transcriptase (RNase H). Second strand cDNA synthesis was subsequently performed using DNA Polymerase I and RNase H. Double-stranded cDNA was purified using AMPure XP beads. Remaining overhangs of the purified double-stranded cDNA were converted into blunt ends via exonuclease/polymerase activities. After adenylation of 3′ ends of DNA fragments, NEBNext Adaptor with hairpin loop structure was ligated to prepare for hybridization. In order to select cDNA fragments of preferentially 150∼200bp in length, the library fragments were purified with AMPure XP system (Beckman Coulter, Beverly, USA). The libraries were sequenced in Illumina for 20 million reads with pair-end 150 bp (PE150). Downstream analysis was performed using a combination of programs including STAR, HTseq and Cufflink. Alignments were parsed using Tophat program and DEGs were determined through DESeq2/edgeR.

### Quantification and statistical analysis

Data are presented as mean ± SEM. Statistical significance was determined using two-tailed Student’s *t*-test unless otherwise indicated. Sample sizes are indicated in figure legends. Statistical analyses were performed using GraphPad Prism software. Differences were considered significant at *p* < 0.05.

## Supplemental information

Document S1. Figures S1–S8

Data S1. RNA-seq data from interscapular brown adipose tissue of control and Cox10^BKO^ mice, related to Figure 3

Data S2. Uncropped western blots
