# Supplementary material for: Cox10-mediated mitochondrial respiration in brown adipocytes regulates adaptive thermogenesis and systemic metabolism
Source: iScience. 2026 Jun 26;29(7):116442. doi: 10.1016/j.isci.2026.116442 (PMC13377871; doi:10.1016/j.isci.2026.116442)
Supplement: Document S1. Figures S1–S8 [file mmc1.pdf]

iScience, Volume *29*

Supplemental information

Cox10-mediated mitochondrial respiration in brown adipocytes regulates adaptive thermogenesis and systemic metabolism

Esther Paulo, Yuanyuan Wu, and Biao Wang

SUPPLEMENTARY INFORMATION

Supplementary information includes five supplementary figures and one supplementary data file.

Figure S1. Expression of ETC genes is not affected in the iBAT of the Cox10^{BKO} mice. (A) qPCR of mitochondria-encoded ETC genes in the iBAT of ~8-week-old male CON and Cox10^{BKO} mice. (B) qPCR of nuclear-encoded ETC genes in the iBAT of ~8-week-old male CON and Cox10^{BKO} mice. Sample size: CON (n=6) and Cox10^{BKO} (n=5).

Figure S2. CLAMS analysis of the Cox10^{BKO} mice. (A) Average (top) and scatter plots (bottom) of energy expenditure (EE) during night and day in ~8-10-week-old male CON and Cox10^{BKO} mice. (B) Average (top) and scatter plots (bottom) of respiratory exchange ratio (RER) during night and day in ~8-10-week-old male CON and Cox10^{BKO} mice. (C) Average (top) and scatter plots (bottom) of food intake during night and day in ~8-10-week-old male CON and Cox10^{BKO} mice. (D) Average (top) and scatter plots (bottom) of activity during night and day in ~8-10-week-old male CON and Cox10^{BKO} mice. Sample size: CON (n=10) and Cox10^{BKO} (n=11).

Figure S3. Ancillary analysis of RNA-seq data in the Cox10^{BKO} mice. (A) Heatmap showing the log2 fold-change of ETC genes in the BAT of the Cox10^{BKO} mice. (B) Heatmap showing log2 fold changes of known ATF4 target genes in the BAT of the Cox10^{BKO} and Lrpprc^{BKO} mice and in the heart of the cardiomyocyte-specific Lrpprc, Tfam, Polrmt, Twinkle Mterf4 knockout mice. (C) Violin plot showing the log2 fold-change of genes involved in ribosome biogenesis, ribosome proteins, and tRNA aminoacylation in the heart of the cardiomyocyte-specific Lrpprc, Tfam, Polrmt, Twinkle Mterf4 knockout mice.

Figure S4. mTORC1 activity in the Cox10-deficient brown adipocytes**.** (A) Immunoblots of p-4Ebp1, total 4Ebp1, p-S6, total S6, and Hsp90 in wild-type and Cox10-deficient brown adipocytes. (B) Quantifications of the ratio of p-4Ebp1 to total 4Ebp1 and the ratio of p-S6 to total S6 in wild-type and Cox10-deficient brown adipocytes.

Figure S5. Metabolic phenotypes in the Cox10^{BKO} female mice under HFD. (A) Body weight of female CON and Cox10^{BKO} mice under 20 weeks HFD. (B) Body weight, lean and fat mass of female CON and Cox10^{BKO} mice after HFD. (C) Representative images of dissected iWAT, eWAT and iBAT from female CON and Cox10^{BKO} mice after HFD. (D) Tissue mass of eWAT, iWAT, and iBAT of female CON and Cox10^{BKO} mice after HFD. (E) Left: Serum glucose levels during ITT in female CON and Cox10^{BKO} mice after HFD. Right: Area under the curve (AUC) values of glucose levels in first 30 minutes of ITTs shown. Sample size: CON (n=7) and Cox10^{BKO} (n=8). Data was presented as average ± SEM. Student t-test. *: $p<0.05$ and **: $p<0.01$.

Figure S6. Cox10^{BKO} mice exhibited WAT browning at RT. (A) qPCR of thermogenic genes in the iWAT of ~8-week-old male CON and Cox10^{BKO} mice housed at RT. Sample size: CON (n=4) and Cox10^{BKO} (n=4). (B) Representative H&E staining of iWAT from male CON and Cox10^{BKO} mice. Red arrowhead: multilocular beige adipocytes. Scale bar: 100 μm.

Figure S7. Expression of ETC genes is not affected in the iBAT of the Cox10^{BKO} mice housed at thermoneutrality. (A) qPCR of mitochondria-encoded ETC genes in the iBAT of ~8-week-old male CON and Cox10^{BKO} mice after 4-week 30°C housing. (B) qPCR of nuclear-encoded ETC genes in the iBAT of ~8-week-old male CON and Cox10^{BKO} mice after 4-week 30°C housing. Sample size: CON (n=5) and Cox10^{BKO} (n=7).

Figure S8. CLAMS analysis of the Cox10^{BKO} mice housed at thermoneutrality. (A) Recordings of energy expenditure (EE, kcal/hour) in ~8-week-old male CON and Cox10^{BKO} mice for 3 days at 30°C. Red arrowhead: time of CL 316,423 (CL) injection. (B) Average hourly CL-induced EE in the aforementioned experiment. (C) Average (top) and scatter plots (bottom) of energy expenditure (EE) during night and day in ~8-10-week-old male CON and Cox10^{BKO} mice housed at 30°C. (D) Average (top) and scatter plots (bottom) of respiratory exchange ratio (RER) during night and day in ~8-10-week-old male CON and Cox10^{BKO} mice housed at 30°C. (E) Average (top) and scatter plots (bottom) of food intake during night and day in ~8-10-week-old male CON and Cox10^{BKO} mice housed at 30°C. (F) Average (top) and scatter plots

(bottom) of activity during night and day in ~8-10-week-old male CON and $Cox10^{BKO}$ mice housed at 30°C. Sample size: CON (n=4) and $Cox10^{BKO}$ (n=11). Data was presented as average ± SEM. Student t-test. *: $p<0.05$ and **: $p<0.01$.

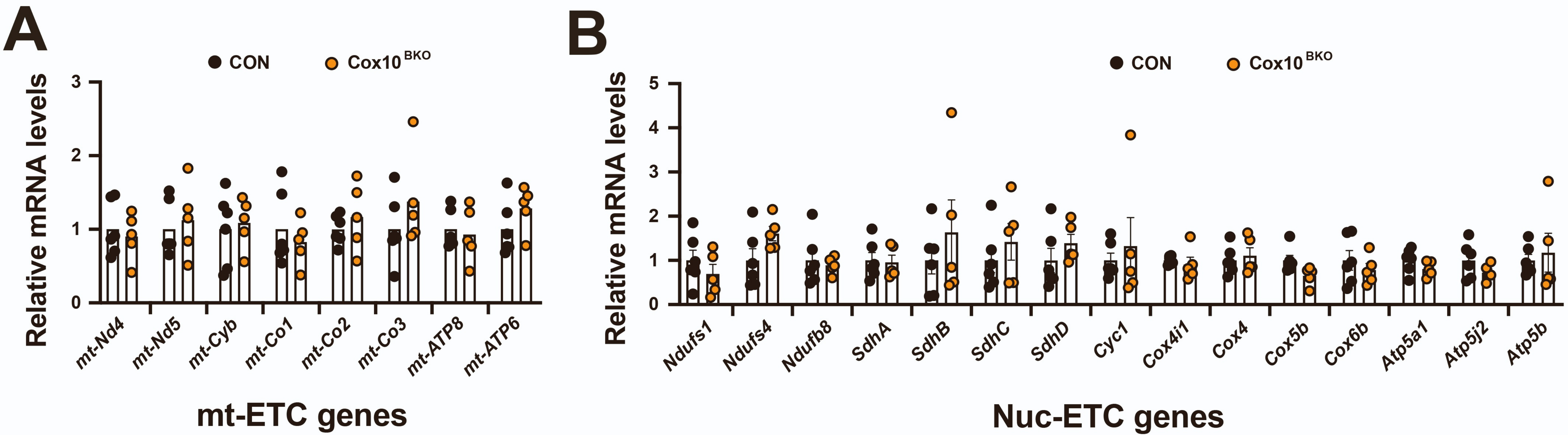

A
CON
Cox10 BKO
Relative mRNA levels
3
2
1
0
mt-Nd4
mt-Nd5
mt-Cyb
mt-Co1
mt-Co2
mt-Co3
mt-ATP8
mt-ATP6
mt-ETC genes
B
CON
Cox10 BKO
Relative mRNA levels
5
4
3
2
1
0
Ndufs1
Ndufs4
Ndufb8
SdhA
SdhB
SdhC
SdhD
Cyc1
Cox4i1
Cox4
Cox5b
Cox6b
Atp5a1
Atp5j2
Atp5b
Nuc-ETC genes

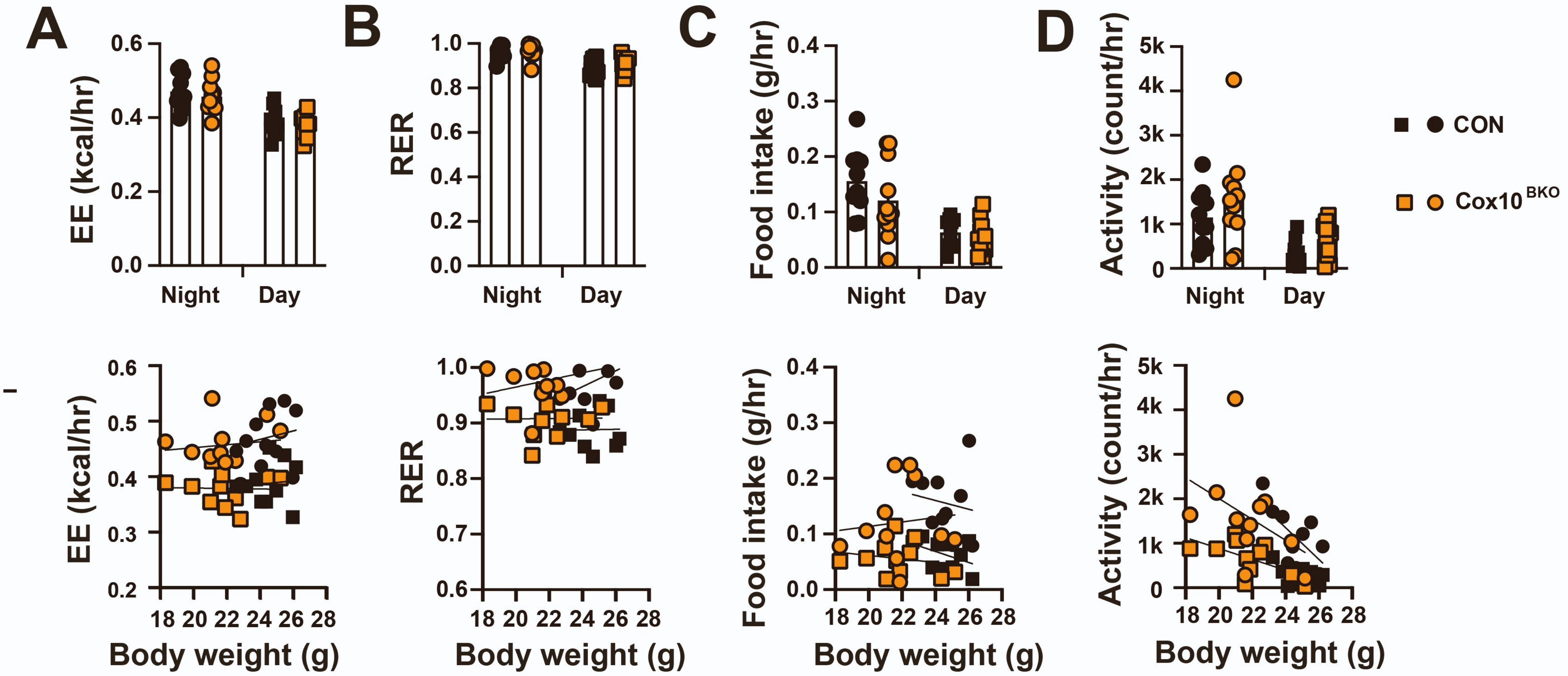

A
B
C
D
EE (kcal/hr)
RER
Food intake (g/hr)
Activity (count/hr)
Night
Day
CON
Cox10 BKO
Body weight (g)

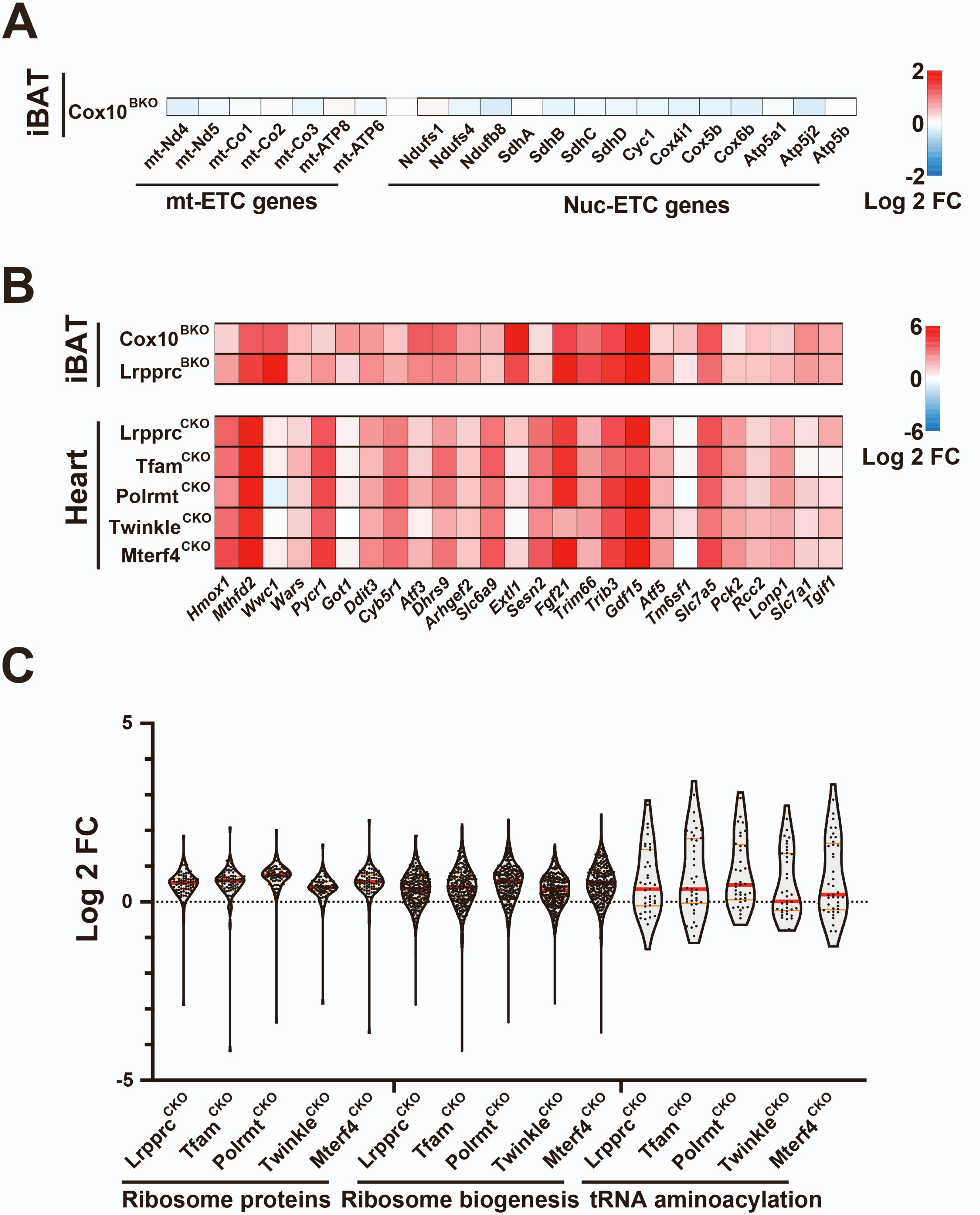
A
iBAT
Cox10 BKO
mt-Nd4
mt-Nd5
mt-Co1
mt-Co2
mt-Co3
mt-ATP8
mt-ATP6
Ndufs1
Ndufs4
Ndufb8
SdhA
SdhB
SdhC
SdhD
Cyc1
Cox4i1
Cox5b
Cox6b
Atp5a1
Atp5j2
Atp5b
mt-ETC genes
Nuc-ETC genes
2
0
-2
Log 2 FC
B
iBAT
Cox10 BKO
Lrpprc BKO
Heart
Lrpprc CKO
Tfam CKO
Polrmt CKO
Twinkle CKO
Mterf4 CKO
6
0
-6
Log 2 FC
Hmox1
Mthfd2
Wwc1
Wars
Pycr1
Got1
Ddit3
Cyb5r1
Atf3
Dhrs9
Arhgef2
Slc6a9
Extl1
Sesn2
Fgf21
Trim66
Trib3
Gdf15
Atf5
Tm6sf1
Slc7a5
Pck2
Rcc2
Lonp1
Slc7a1
Tgif1
C
Log 2 FC
5
0
-5
Lrpprc CKO
Tfam CKO
Polrmt CKO
Twinkle CKO
Mterf4 CKO
Lrpprc CKO
Tfam CKO
Polrmt CKO
Twinkle CKO
Mterf4 CKO
Lrpprc CKO
Tfam CKO
Polrmt CKO
Twinkle CKO
Mterf4 CKO
Ribosome proteins
Ribosome biogenesis
tRNA aminoacylation

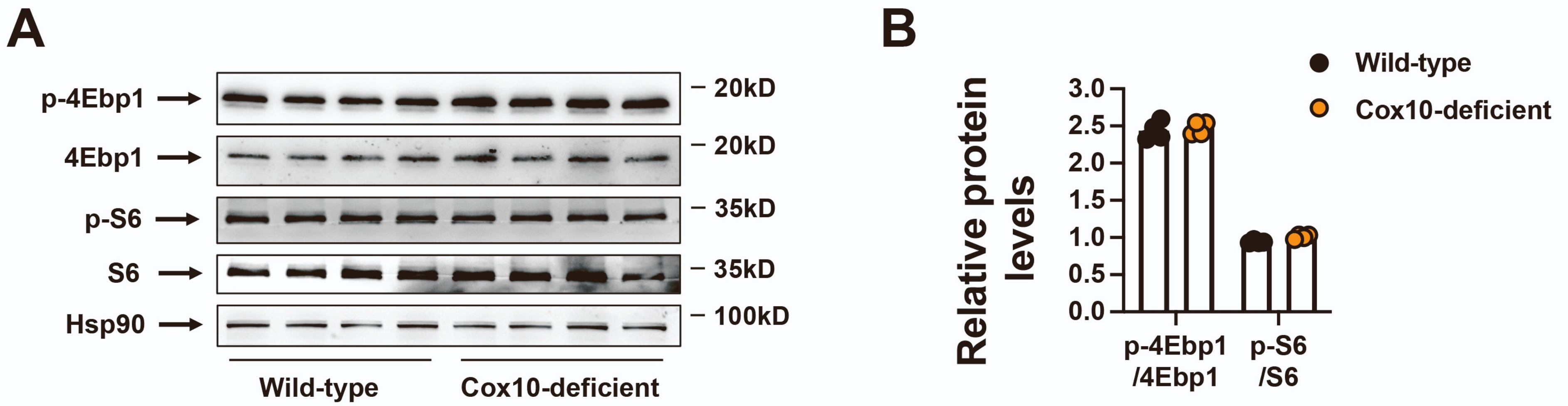
A
p-4Ebp1
4Ebp1
p-S6
S6
Hsp90
20kD
20kD
35kD
35kD
100kD
Wild-type
Cox10-deficient
B
Wild-type
Cox10-deficient
Relative protein levels
3.0
2.5
2.0
1.5
1.0
0.5
0.0
p-4Ebp1 /4Ebp1
p-S6 /S6

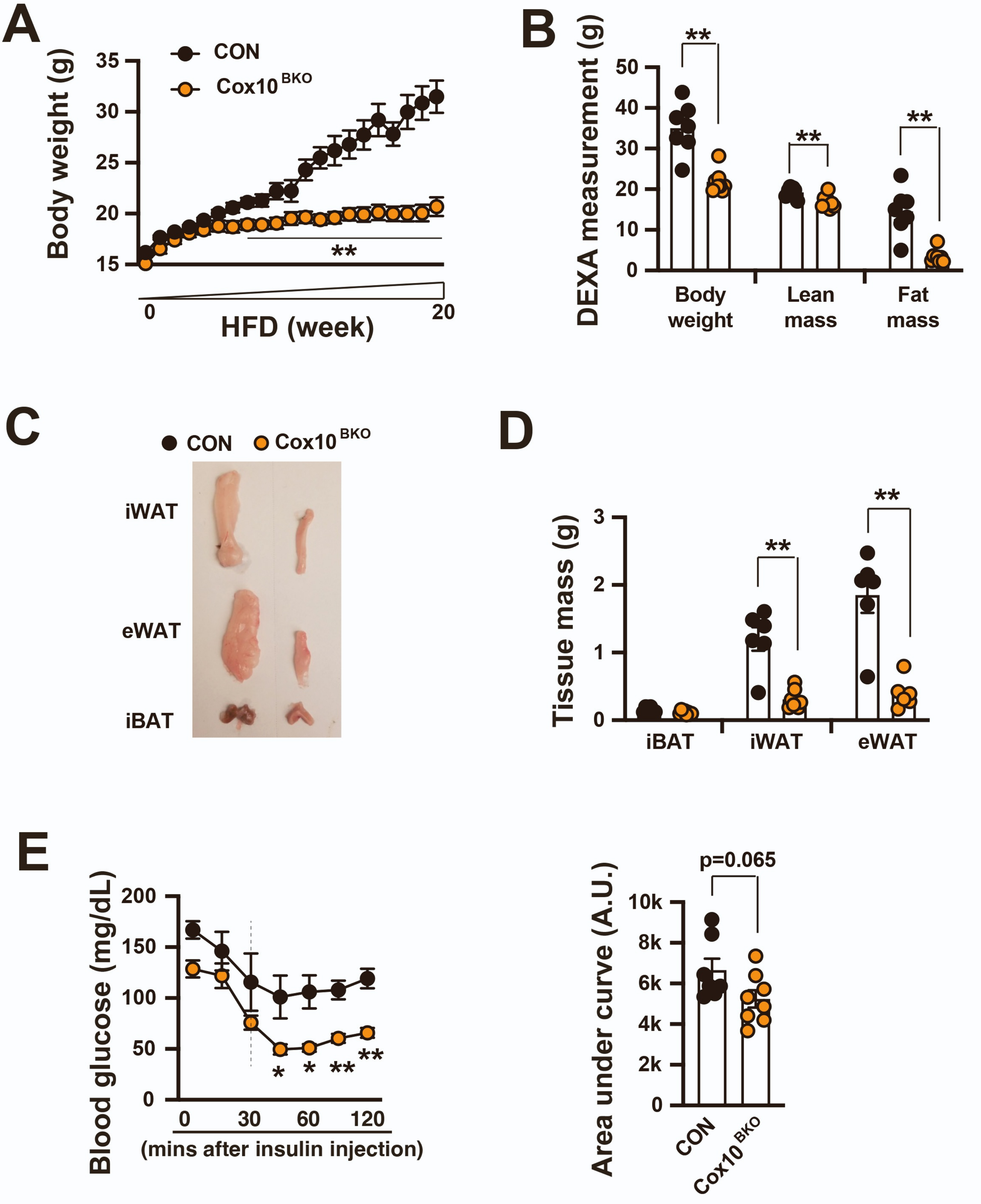

A
CON
Cox10 BKO
Body weight (g)
35
30
25
20
15
**
0
20
HFD (week)
B
DEXA measurement (g)
50
40
30
20
10
0
**
**
**
Body weight
Lean mass
Fat mass
C
CON
Cox10 BKO
iWAT
eWAT
iBAT
D
Tissue mass (g)
3
2
1
0
**
**
iBAT
iWAT
eWAT
E
Blood glucose (mg/dL)
200
150
100
50
0
0
30
60
120
(mins after insulin injection)
*
*
**
**
Area under curve (A.U.)
p=0.065
10k
8k
6k
4k
2k
0
CON
Cox10 BKO

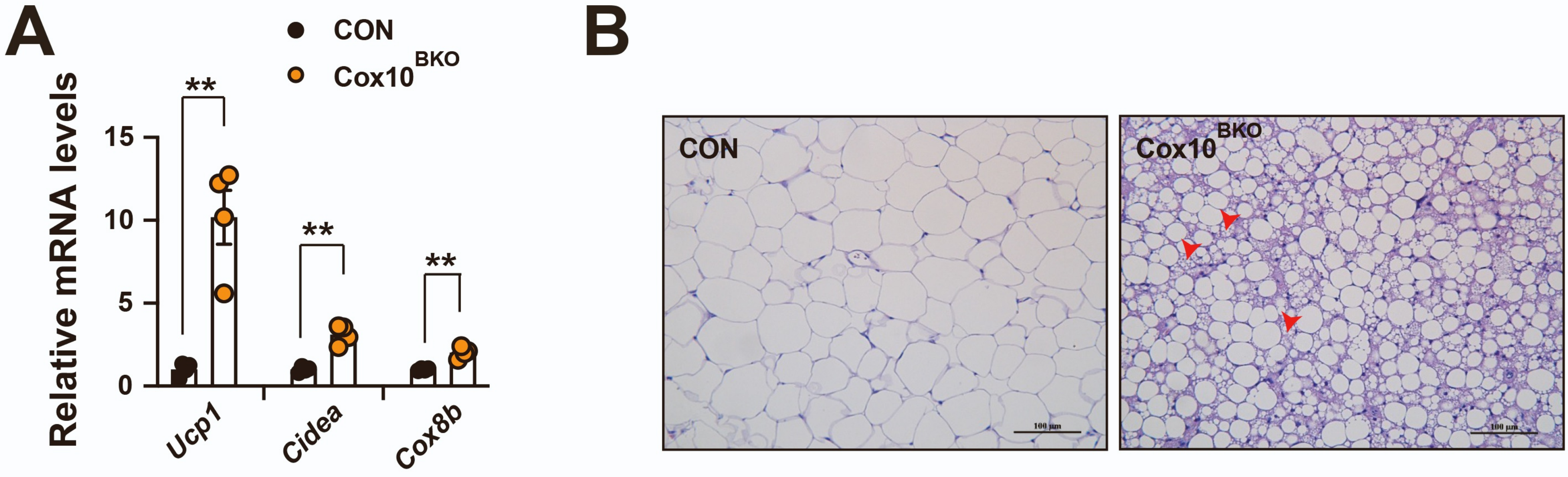

A
CON
Cox10 BKO
Relative mRNA levels
15
10
5
0
**
**
**
Ucp1
Cidea
Cox8b
B
CON
Cox10 BKO
100 μm
100 μm

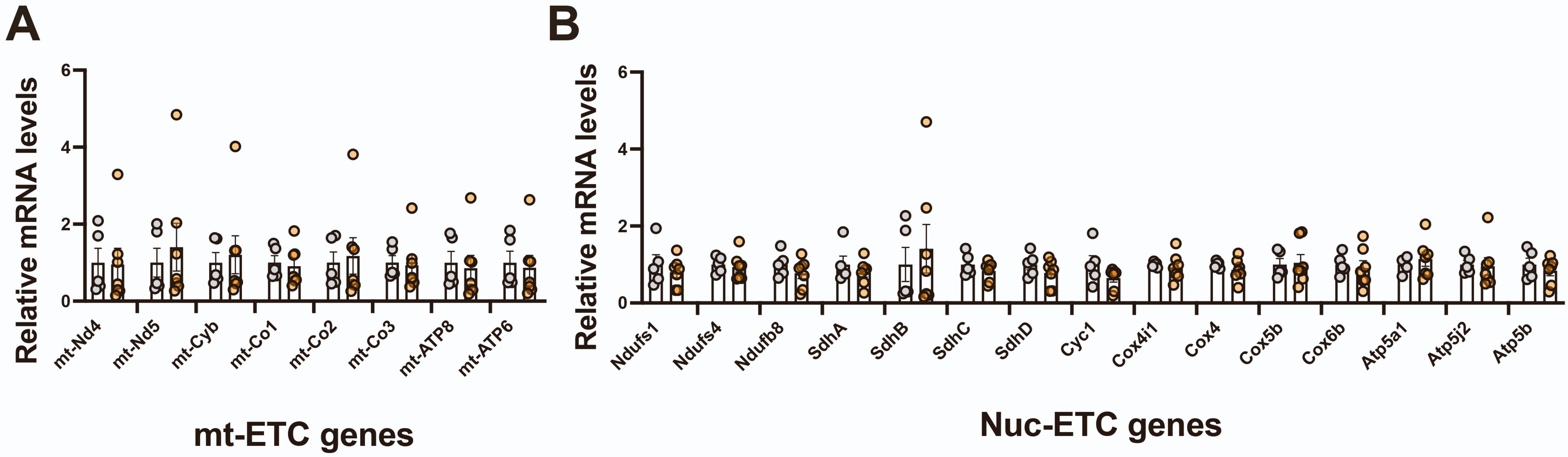
A
B
Relative mRNA levels
6
4
2
0
mt-Nd4
mt-Nd5
mt-Cyb
mt-Co1
mt-Co2
mt-Co3
mt-ATP8
mt-ATP6
mt-ETC genes
Ndufs1
Ndufs4
Ndufb8
SdhA
SdhB
SdhC
SdhD
Cyc1
Cox4i1
Cox4
Cox5b
Cox6b
Atp5a1
Atp5j2
Atp5b
Nuc-ETC genes

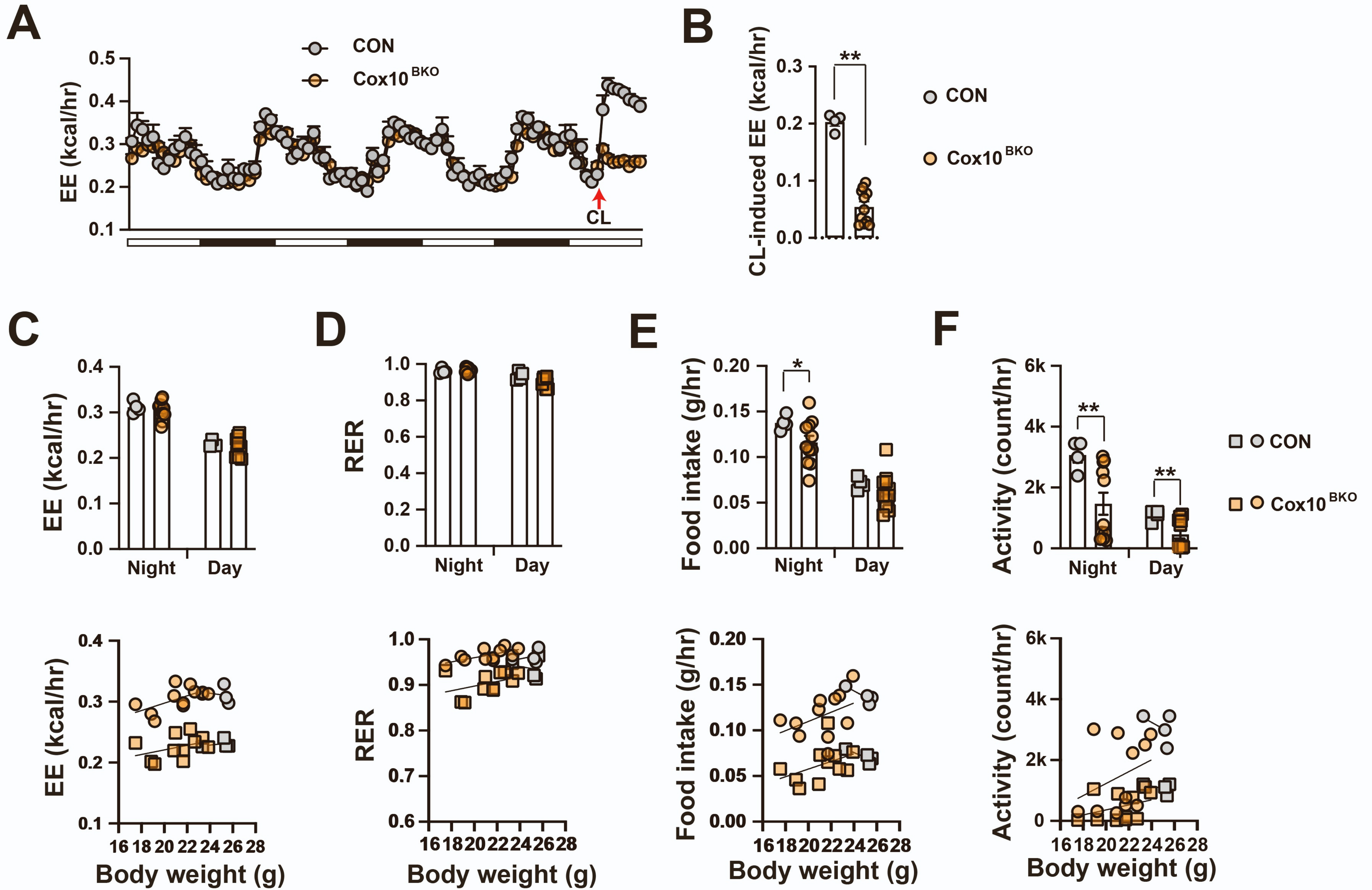
A
B
C
D
E
F
CON
Cox10 BKO
EE (kcal/hr)
CL
CL-induced EE (kcal/hr)
**
RER
Food intake (g/hr)
*
Activity (count/hr)
Night
Day
Body weight (g)
