# Supplementary material for: Cox10-mediated mitochondrial respiration in brown adipocytes regulates adaptive thermogenesis and systemic metabolism
Source: iScience. 2026 Jun 26;29(7):116442. doi: 10.1016/j.isci.2026.116442 (PMC13377871; doi:10.1016/j.isci.2026.116442)

Fig 1B

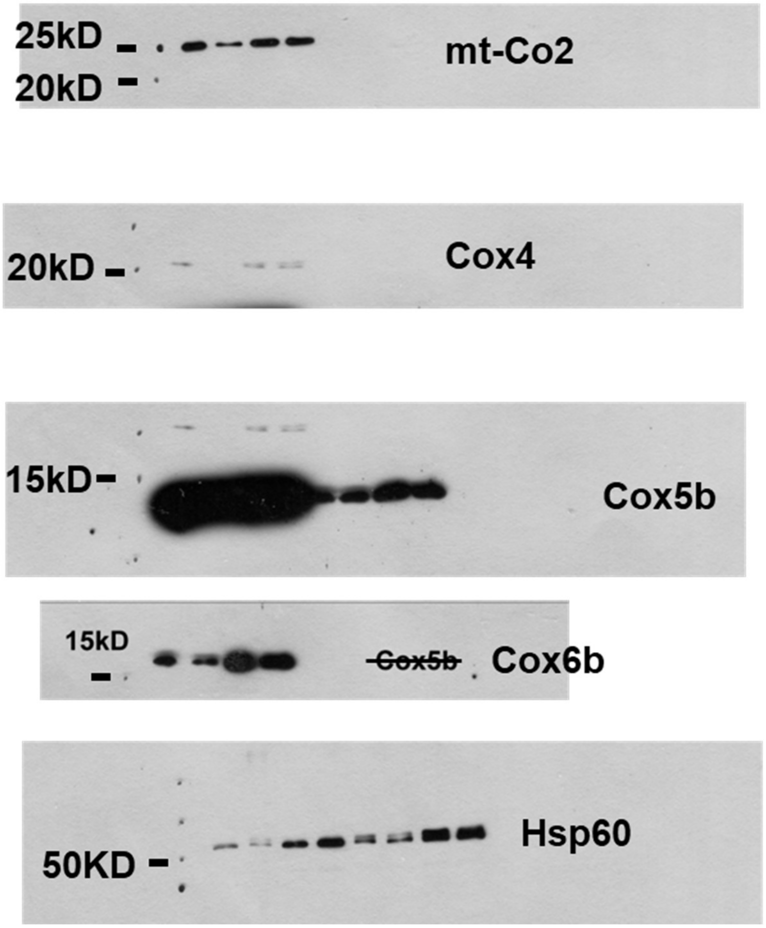

25kD
20kD
mt-Co2
20kD
Cox4
15kD
Cox5b
15kD
Cox5b
Cox6b
50KD
Hsp60


Fig 1C

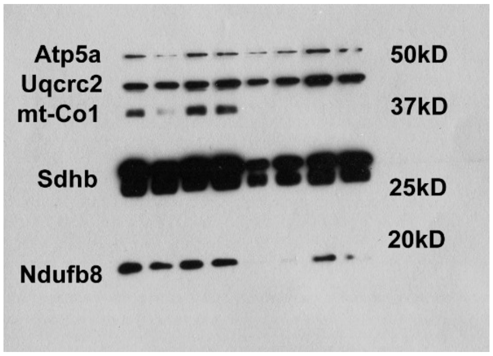

Atp5a
Uqcrc2
mt-Co1
Sdhb
Ndufb8
50kD
37kD
25kD
20kD

Fig 3B

✓ Biological repeat1: four bands from the same membrane

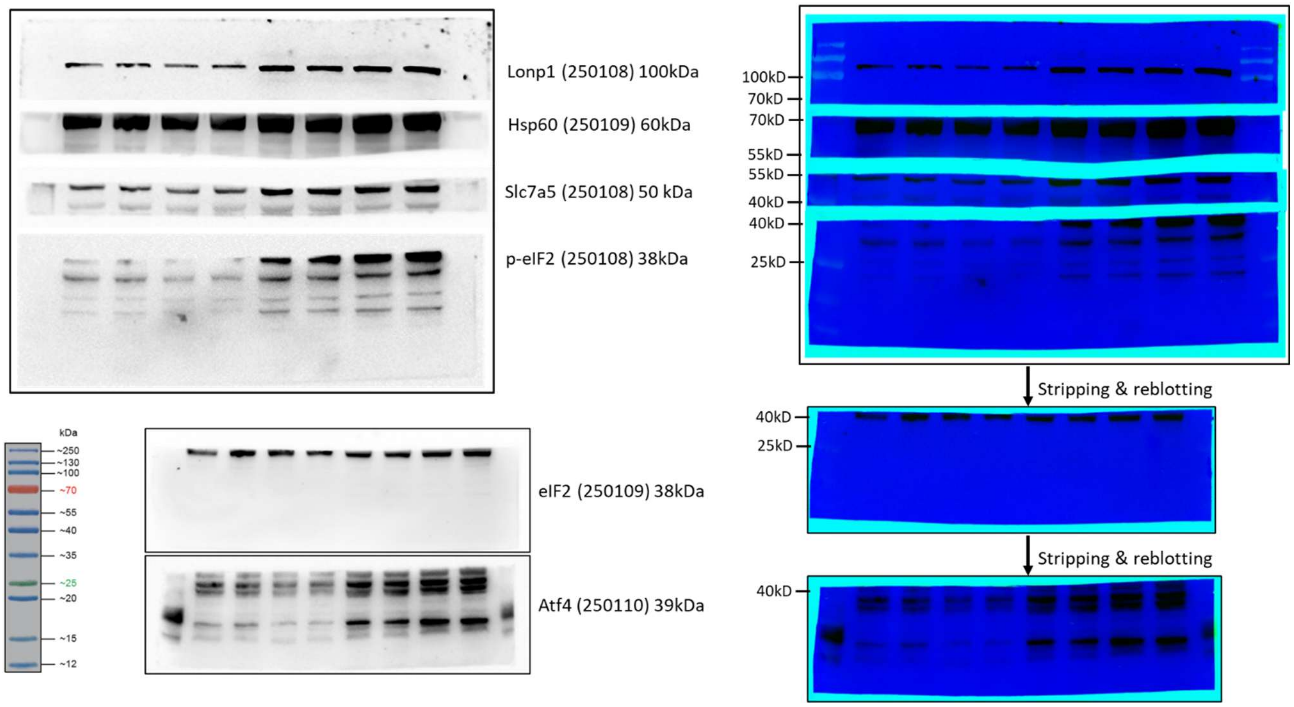

**Fig4F**

- ✓ Biological repeat1:
  - ○ Puromycylated proteins (241112)

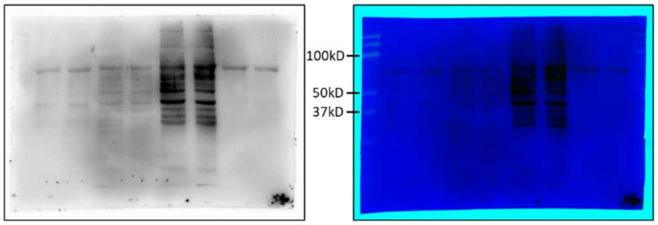


- ○ Hsp90 (241115)

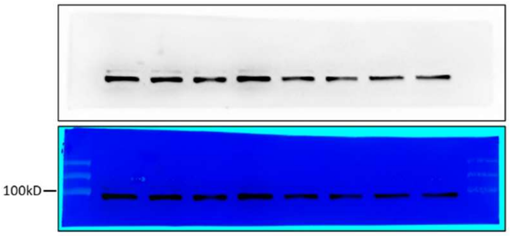


- ✓ Biological repeat2:
  - ○ Puromycylated proteins (241004)

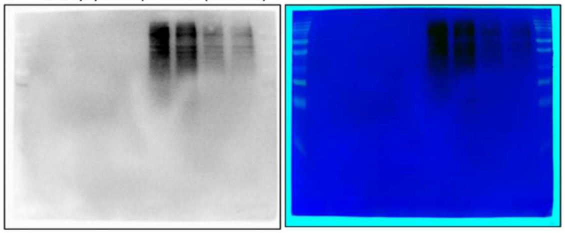

- ○ Hsp90 (241005)

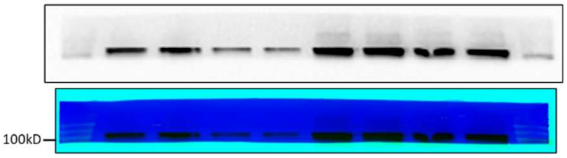


- ✓ Biological repeat3:
  - ○ Puromycylated proteins (241023)

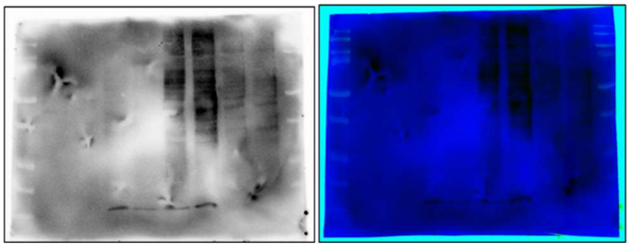

- ○ Hsp90 (241024)

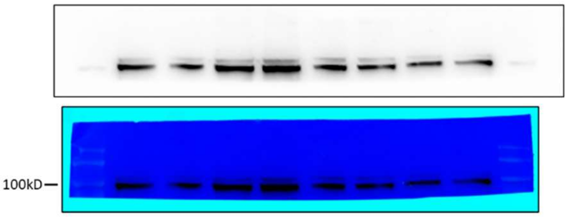

**Fig 6A**

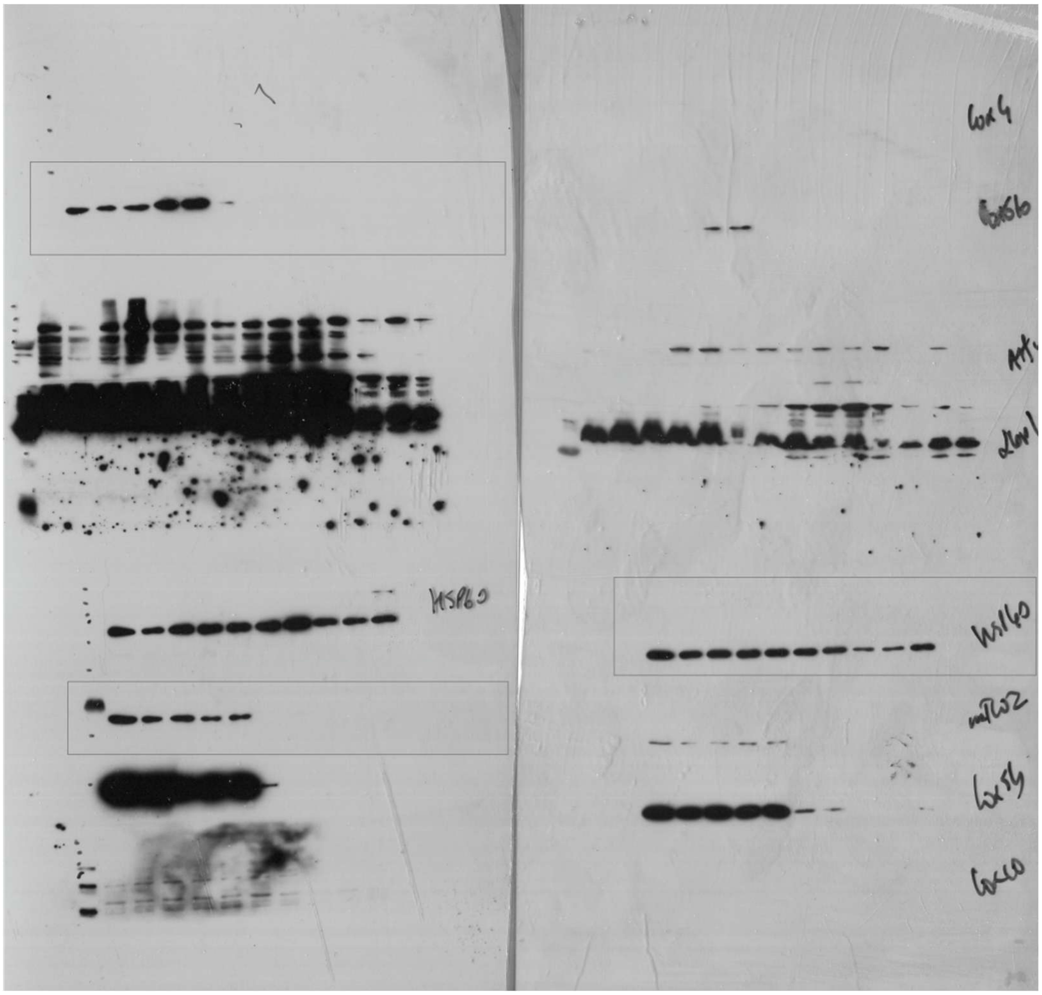

**SFig4A**

- ✓ Biological repeat1:

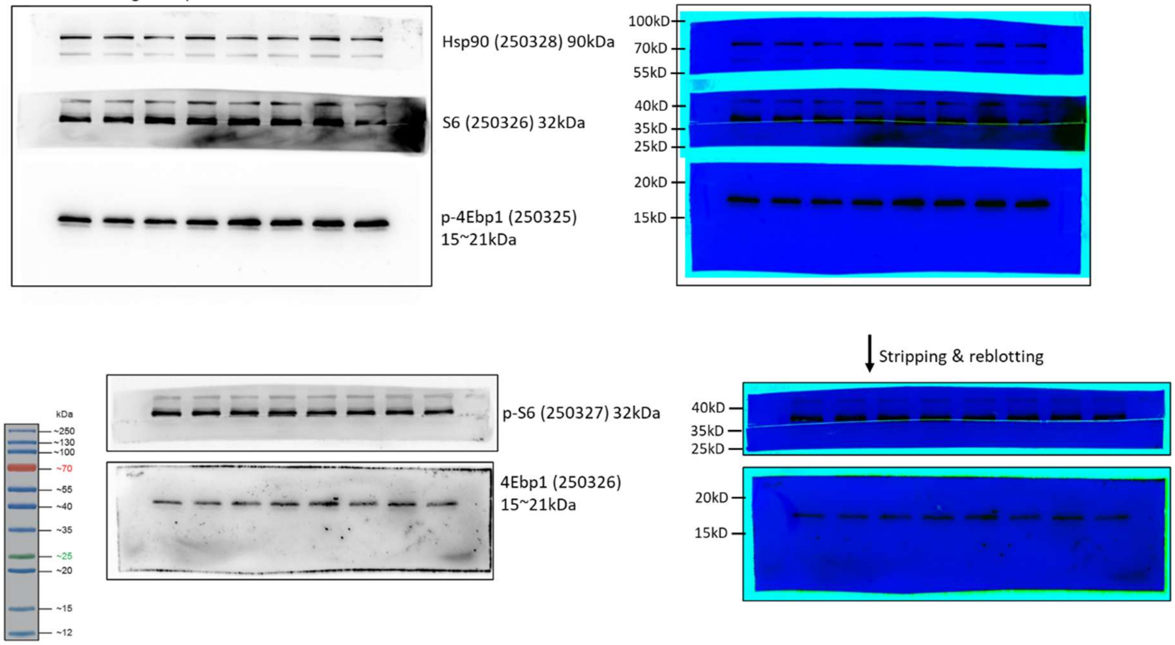

Supplement: Data S2. Uncropped western blots [file mmc3.pdf]
